# Genetic Testing in the Management of Adult CKD

**DOI:** 10.1681/ASN.0000000913

**Published:** 2025-10-29

**Authors:** Fouad T. Chebib, Xiangling Wang, Suneel M. Udani, Maggie Westemeyer, Dinah Clark, Zhiji Zhang, Michelle S. Bloom, Hila Milo Rasouly, Victoria Kolupaeva, Mohammad R. Mizani, Neville R. Dossabhoy, Arman Faravardeh, Zachary P. Demko, Sri Kotte, Sumit Punj, Steven L. Chapman, Matthew Rabinowitz, Ronen Schneider, Hossein Tabriziani, Sangeeta Bhorade, Ali G. Gharavi, Neera K. Dahl

**Affiliations:** 1 Division of Nephrology and Hypertension, Mayo Clinic, Jacksonville, Florida; 2 Department of Kidney Medicine, Center for Personalized Genetic Healthcare, Cleveland Clinic, Cleveland, Ohio; 3 Nephrology Associates of Northern Illinois and Indiana, Chicago, Illinois; 4 Natera, Inc., Austin, Texas; 5 Division of Nephrology, Department of Medicine, Columbia University Vagelos College of Physicians and Surgeons, New York, New York; 6 South Texas Renal Care Group, San Antonio, Texas; 7 Division of Nephrology, University of Mississippi Medical Center, Jackson, Mississippi; 8 SHARP Kidney and Pancreas Transplant Center, San Diego, California; 9 Division of Nephrology and Hypertension, Mayo Clinic, Rochester, Minnesota

**Keywords:** CKD, genetic renal disease, interventional nephrology, kidney

## Abstract

**Key Points:**

- Genetic testing in CKD showed diagnostic and clinical utility in the year following reporting of test results.
- Genetic testing was helpful for or changed management in 86% of patients with a positive finding and 42% with a negative finding.
- Clinical utility was seen across 12 clinical cohorts, including CKD of unknown etiology and hypertension or diabetes-related nephropathy.

**Background:**

CKD is a significant public health burden, affecting >800 million people worldwide with significant cost to the health care system. CKD is a disease process with substantial genetic and phenotypic heterogeneity that can obscure a definitive diagnosis, resulting in suboptimal management. Recent guidelines support greater adoption of genetic testing in CKD. We assessed the diagnostic and clinical utility in the year following broad-panel CKD genetic testing.

**Methods:**

The Renasight Clinical Application, Review, and Evaluation (RenaCARE) study (NCT05846113) was a single-arm, interventional, prospective, multicenter study evaluating the utility of genetic testing with a 385-gene panel on the diagnosis and management of CKD. Clinical history was collected before testing, and nephrologists responded to questionnaires at both 1 month and 1 year after testing. The impact of genetic testing on CKD diagnosis and management were assessed in active study patients at the 1-year time point.

**Results:**

In a cohort of 1388 CKD patients with 13 pretest clinical categories of CKD, 335 (24%) patients had a positive genetic finding; 1174 had a questionnaire completed at 1 year and were included in the analysis. Genetic testing was reported helpful for clinical management and/or led to a change in management in 86% of patients with a positive test finding and 42% of patients with a negative test finding. In addition, genetic testing resulted in a change in the physician estimation of the 5-year prognosis for 55% of patients with a positive test finding and 18% of those with a negative test finding.

**Conclusions:**

Supporting the previous results of the RenaCARE study at 1 month, this report demonstrated that genetic testing was helpful in the clinical management and estimated prognosis of patients with CKD.

**Clinical Trial registry name and registration number:**

ClinicalTrials.gov, NCT05846113.

## Visual Abstract

**Figure:**
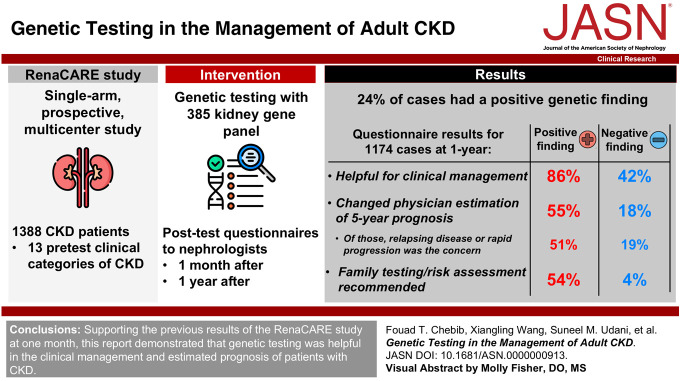

## Introduction

CKD is a significant public health burden, affecting more than 800 million people worldwide.^1^ Encompassing dozens of distinct conditions that affect kidney function, CKD poses a challenge to medical management because of the heterogeneity of the underlying disease pathology and the vast spectrum of clinical phenotypes that may present.^2^ As such, providing an accurate diagnosis of the underlying condition remains an unmet need.

Genetic testing, long a mainstay in fields such as oncology, has recently expanded into the realm of nephrology. Over the past decade, numerous guidelines have advocated for genetic testing across specific CKD phenotypes.^3–7^ In 2024, the Kidney Disease Improving Global Outcomes (KDIGO) organization and the National Kidney Foundation (NKF) issued pivotal recommendations endorsing widespread genetic testing within the CKD population.^8–11^ KDIGO highlighted the critical role of genetics in classifying and managing CKD, recommending genetic testing to enhance diagnostic accuracy and enable personalized treatment strategies.^10^ The NKF working group used a modified Delphi process to develop algorithms and consensus statements to advance testing in kidney disease and issued a general recommendation advocating genetic testing in cases of kidney-related abnormalities where genetic etiology is considered, following comprehensive clinical evaluation.^9^

The clinical utility of broad-panel and whole-exome genetic testing in populations with CKD has been well-established.^12–26^ Numerous studies have demonstrated significant improvement in a variety of outcomes, such as disease reclassification, optimization of existing therapies, facilitation of referrals to clinical trials, reduction or diagnostic assistance in biopsies, referrals to specialty clinics for managing extrarenal manifestations, and determination of prognosis for transplant referrals, all contributing to demonstrated economic benefits. However, these studies often had limitations, including a focus on specific clinical phenotypes, narrow population demographics, limited cohort sizes, or constrained timelines for outcome collection.

The Renasight Clinical Application, Review, and Evaluation (RenaCARE) study was designed to evaluate the utility of genetic testing across a broad CKD population, enrolling over 1700 patients comprising more than a dozen different CKD phenotypes at 31 diverse community and academic centers throughout the United States. Our initial publication from the RenaCARE study documented the diagnostic yield and diagnostic utility of broad genetic testing in CKD within the first month post-testing, and provided an overview of its clinical utility.^27^ In this study, we delve deeper into the management changes reported by nephrologists over the year after testing. We assess the specific diagnostic, management, and treatment implications, expanding the discussion on the clinical utility of genetic testing in CKD. This includes a detailed examination of the management impacts within specific clinical cohorts, analysis of key subgroups such as patients with multiple gene findings, those who underwent complementary biopsy, those with transplant implications, and the exploration of US Food and Drug Administration–approved therapies and clinical trial enrollment options.

## Methods

### Study Design and Participants

The RenaCARE study (ClinicalTrials.gov NCT05846113) was an open label, interventional, single-arm, unblinded, prospective, multicenter study. It was designed to assess the diagnostic and clinical utility of genetic testing in patients with CKD using a medical exome-based 385-kidney gene panel (the Renasight [Renasight is a Natera-owned trademark] test, Natera, Inc., Austin, TX). As previously reported, adults (aged ≥18 years) diagnosed with CKD and treated at 31 academic or community practices across the United States were enrolled.^27^ Eligible patients included those with a CKD diagnosis and/or those who fell into one of 13 clinical kidney disease categories (Supplemental Table 1). Patients with a confirmed nongenetic etiology for their CKD were excluded. All participants provided informed consent under a local institutional review board–approved protocol. On enrollment, each patient underwent genetic testing with the Renasight test, and the results were shared with the participating nephrologists for use in clinical decision making. To ensure representation of all clinical disease categories, enrollment was capped for categories that exceeded 10% of the total cohort, per the study protocol. In accordance with this, enrollment of patients categorized with cystic nephropathies, nephropathies associated with hypertension, or nephropathies associated with diabetes mellitus was capped. The cohort of patients who remained active in the study at 1 year were used to determine the diagnostic and clinical utility implications.

Demographic information, clinical data, and medical history were documented at baseline, before testing, by clinical research coordinators.^27^ Follow-up questionnaires were issued to the referring nephrologists both 1 month and 1 year after results were reported, querying the actions taken subsequent to receipt of the genetic test results, and the reasoning behind those actions. The primary outcomes of this analysis, involving the subcohort of patients with a completed 1-year questionnaire, focused on the impact of genetic testing on patient management as reported by the 1-year post-test questionnaire (Supplemental Table 2). Patients with a positive response (done because of genetic test results) to question 4a–g, j–l, n–q were considered to have had a change in management because of genetic testing. The study adhered to the ethical standards of the Declaration of Helsinki.

### Renasight Next-Generation Sequencing Panel Sequencing, Data Analysis Variant Interpretation

Genomic material obtained from blood or saliva swab underwent library preparation and sequencing following previously established protocols.^28^ Variants were assessed and classified based on a standardized operating procedure that adheres to the American College of Medical Genetics and Genomics and Association for Molecular Pathology guidelines for sequence variant interpretation.^27–29^ Positive findings included (*1*) a monoallelic pathogenic (P)/likely pathogenic (LP) variant in a gene associated with an autosomal dominant or X-linked disorder, (*2*) biallelic P/LP variants in a gene associated with an autosomal recessive disorder, and/or (*3*) biallelic *APOL1* G1 and/or G2 high-risk alleles. Negative findings included (*1*) monoallelic P/LP variants in a gene associated with an autosomal recessive disorder (carriers), (*2*) variants of uncertain significance, and (*3*) no P/LP variants detected. The 385 genes tested on the Renasight panel all target monogenic diseases, and risk alleles for *APOL1*.^30^

### Statistical Methods

Descriptive statistical analyses were conducted to summarize the demographics, clinical kidney disease categorizations, and 1-year follow-up data of the cohort. Comparative analyses between patients with positive and negative gene findings were conducted using two-sided Fisher exact tests for categorical variables and the Wilcoxon rank-sum test for continuous variables. To control the type 1 error rate from multiple comparisons, the Benjamini–Hochberg false discovery rate (FDR) method^31^ was applied to adjust *P* values from tests involving related hypotheses, such as comparisons in demographics and in management change, respectively. Statistical significance was defined as *P* < 0.05 after FDR correction. The same analyses were performed in the overall 1-year study cohort and in each clinical kidney disease category, respectively. Means are presented with SD.

Missing data from participants who did not complete the 1-year questionnaire were minimal (<15%), and these individuals were not significantly different from those who completed the questionnaire with respect to baseline characteristics and Renasight test results (*P* > 0.05). Therefore, the missing data were assumed to be missing at random, and analyses were conducted using a complete-case approach without imputation.

## Results

### Demographics of the Clinical Cohort

**Figure 1:**
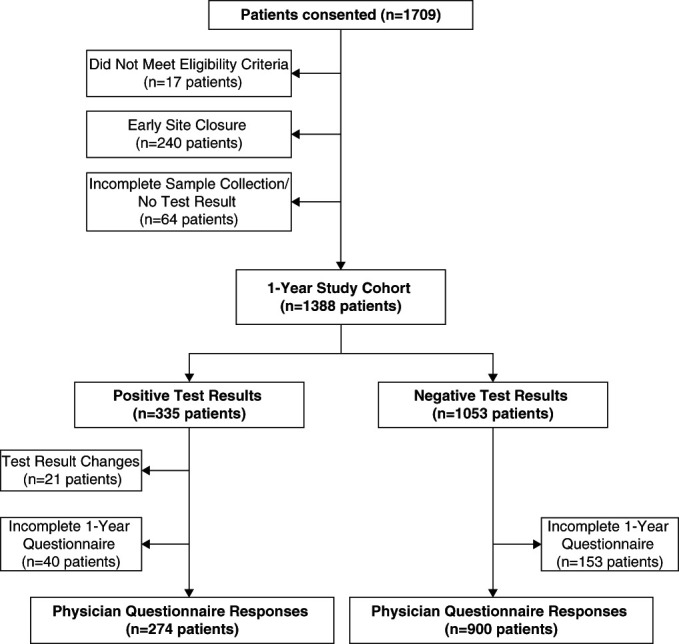
Consolidated Standards of Reporting Trials (CONSORT) diagram.

The RenaCARE study enrolled a total of 1709 patients,^27^ between May 2021 and May 2022, with 81% (*n*=1388) remaining active in the study at the 1-year follow-up time point (Figure 1 and Supplemental Table 3) Of these patients, 24% (*n*=335) received a positive gene finding. This included 21 patients whose initial negative test result (including variants of uncertain significance) was later reclassified as positive, accounting for 6% of all positive cases (Supplemental Material). At the 1-year follow-up, the questionnaire assessing the effect of genetic testing on clinical management was completed for 86% (1191/1388) of the patients, among whom 291 had a positive test result and 900 had a negative test result (Figure 1). Twenty-one patients with upgraded test results (17 with completed questionnaires) were excluded for this analysis because of varying timelines between result upgrade and questionnaire completion, yielding an analysis cohort of 1174 patients, of whom 274 had a positive result. The mean age at the time of testing was 51 years (SD 14), with a nearly equal distribution of sex at birth (female: 49%, male 51%). The cohort was predominantly White (66%), followed by Black (16%) and Asian (6%). Patients with positive gene findings had a mean age of 46 years (SD 14), compared with 52 (SD 14) for those with negative results. A significantly larger proportion of patients with a positive gene finding reported a family history of CKD (46%) compared with those with a negative result (29%; FDR-adjusted *P* < 0.001; Table 1).

**Table 1:** Demographic characteristics of patients who underwent genetic testing for kidney disease and had follow-up questionnaire at 1 year

Characteristic	All Patients (*N*=1174)	Positive Gene Findings (*n*=274)	Negative Gene Findings (*n*=900)	FDR-Adjusted *P* Value^a^
**Age, yr**				<0.001
Mean (SD)	51 (14)	46 (14)	52 (14)	
Median (range)	53 (18–87)	47 (18–84)	54 (18–87)	
**Age groups, yr**				
18–39	257 (22%)	83 (30%)	174 (19%)	<0.001
40–64	726 (62%)	157 (57%)	569 (63%)	0.13
65+	191 (16%)	34 (12%)	157 (17%)	0.09
**Sex at birth**				0.03
Female	580 (49%)	154 (56%)	426 (47%)	
Male	594 (51%)	120 (44%)	474 (53%)	
**Race**				
Asian	66 (6%)	12 (4%)	54 (6%)	0.4
Black	190 (16%)	57 (21%)	133 (15%)	0.04
Native/Alaskan/Hawaiian/Pacific	14 (1%)	1 (0.4%)	13 (1%)	0.3
Other/unknown/not reported	140 (12%)	29 (9%)	114 (13%)	0.2
White	776 (66%)	180 (66%)	596 (66%)	0.9
**Ethnicity**				0.003
Hispanic or Latino	298 (26%)	49 (18%)	249 (28%)	
Not Hispanic or Latino	857 (74%)	220 (82%)	637 (72%)	
Unknown	19	5	14	
**Family** history **of CKD**				<0.001
No	797 (68%)	149 (5%)	638 (71%)	
Yes	382 (33%)	125 (46%)	257 (29%)	
Not reported	5 (0.4%)	0 (0.0%)	5 (0.5%)	

FDR, false discovery rate.

a False discovery rate correction for multiple testing.

### Diagnostic and Clinical Implications

In total, 335 patients in 12 of 13 pretest clinical diagnostic categories had positive gene findings that spanned 51 genes (Table 2). Positive gene findings in this study have well-established diagnostic implications, as summarized in Table 3. Selected genes from the cohort are listed in Table 4, along with details illustrating how these findings may inform clinical management decisions or guide the selection of appropriate treatment strategies following a definitive molecular diagnosis.

**Table 2:** Pretest clinical diagnostic categories and reported positive gene findings

Pretest Clinical Diagnosis	Number of Patients with Positive Findings	Positive Gene Findings^a^ (Number of Times Represented)
Cystic nephropathy	144	*PKD1* (93), *PKD2* (29), *IFT140* (6), *COL4A4* (4), *COL4A3* (3), *SLC7A9* (3), *ALG8* (2), *OFD1* (2), *ABCC8* (1), *ALG9* (1), *APOL1* (1), *COL4A5* (1), *CYP17A1*^b^ (1), *DHCR7* (1), *PRKCSH* (1), *TRPC6* (1)
Proteinuric disease suggestive of a primary glomerulopathy	42	*APOL1* (20), *COL4A4* (7), *COL4A3* (4), *COL4A5* (4), *HBB*^b^ (3), *ABCC8* (1), *CFI* (1), *NPHS2* (1), *ROBO2* (1), *RRM2B* (1), *SMARCAL1* (1), *WAS* (1), *WT1* (1)
Nephropathy associated with hypertension	26	*APOL1* (12), *COL4A3* (2), *COL4A4* (2), *HNF1A* (2), *TTR* (2), *CFI* (1), *COL4A1* (1), *IFT140* (1), *NPHP1* (1), *PKD1* (1), *PKD2* (1), *PROKR2* (1), *SLC2A9* (1), *SLC3A1* (1), *SMAD9* (1)
Kidney failure	26	*APOL1* (9), *TTR* (4), *COL4A3* (3), *COL4A4* (2), *PKD2* (2), *CFH* (1), *CLCN5* (1), *HNF1A* (1), *MAFB* (1), *MC4R* (1), *PKD1* (1), *SLC4A1* (1), *SLC7A9* (1)
CKD of unknown etiology	25	*APOL1* (6), *UMOD* (4), *COL4A5* (2), *SALL1* (2), *SLC7A9* (2), *ATP7B* (1), *CASR* (1), *CLCNKB* (1), *COL4A3* (1), *COL4A4* (1), *CUBN* (1), *GNAS* (1), *HBB*^b^ (1), *IFT140* (1), *NPHP1* (1), *WT1* (1)
Nephropathy associated with diabetes mellitus	16	*APOL1* (4), *COL4A3* (2), *TTR* (2), *ABCC8* (1), *ALG8* (1), *HNF1A* (1), *IFT140* (1), *LMNA* (1), *PKD1* (1), *SCNN1B* (1), *SLC7A9* (1)
Hematuria	15	*COL4A4* (5), *COL4A5* (4), *COL4A3* (3), *ALG9* (1), *COL4A1* (1), *CUBN* (1)
Electrolyte and/or acid–base disorder	11	*SLC12A3* (4), *CLCNKB* (3), *SLC4A1* (2), *CASR* (1), *COL4A1* (1), *COL4A3* (1), *PKD2* (1)
Congenital nephropathy	8	*COL4A5* (5), *COL4A3* (1), *PAX2* (1), *SLC3A1* (1)
Tubulointerstitial disease of unknown etiology	6	*CLCNKB* (1), *COL4A3* (1), *COL4A4* (1), *FAN1* (1), *KANSL1* (1), *NPHP1* (1), *SLC12A3* (1)
Early onset, severe, or familial hypertension	5	*APOL1* (4), *ABCC8* (1), *COL4A4* (1), *COL4A5* (1)
Nephrolithiasis with family history	4	*APOL1* (1), *COL4A4* (1), *KLHL3* (1), *SLC3A1*^b^ (1)
Thrombotic microangiopathy	0	–

a Positive findings are defined as heterozygote/monoallelic for autosomal dominant and X-linked conditions and biallelic for autosomal recessive conditions. For genes that can be both autosomal recessive and autosomal dominant, unless otherwise noted, these variants were monoallelic.

b These gene variants were biallelic.

**Table 3:** Diagnostic implications of positive findings by gene

Gene(s)	Clinical Disease	Diagnostic Implication	# Cases
*PKD1, PKD2*	ADPKD^32^	Prognosis clarification based on gene and mutation type; evaluation of aortic aneurysm and cardiac valve abnormalities, screening for intracranial aneurysm	131
*COL4A3/4/5*	Alport syndrome^3^	Diagnose the etiology hematuria and glomerular disease; evaluate for ocular abnormalities and hearing loss	61
*APOL1*	Susceptibility to kidney failure and focal segmental glomerulosclerosis^41^	May explain etiology of glomerular disease; may explain progression of CKD in individuals with risk factors associated with AMKD	59
*IFT140* ^42^ */ALG8/ALG9* ^43^	ADPKD	Clarification of prognosis to atypical cystic disease	14
*UMOD*	Autosomal dominant tubulointerstitial kidney disease^44^	Diagnose CKD of unknown etiology and/or nonspecific biopsy findings	4
*COL4A1*	*COL4A1*-related disorders^45^ (HANAC; brain small vessel disease with hemorrhage)	Evaluation for aneurysm/stroke, arterial retinal tortuosity, vision loss, other eye disease, heart disease including arrhythmia	3
*NPHP1*	Nephronophthisis^33^	Evaluation and management of liver fibrosis; retinal dystrophy and other eye abnormalities; anomalies of liver, bile duct, spleen, and/or pancreas; structural cardiac defects; structural brain abnormalities	3
*CUBN*	Chronic benign proteinuria^46^	Diagnose the etiology of proteinuria in the setting of retained kidney function	2
*WT1*	*WT1*-related disorders^47^ (Frasier syndrome; Denys–Drash syndrome; Meacham syndrome)	Evaluate for structural urogenital abnormalities, endocrine abnormalities, and screening for germ cell tumors Diagnose the etiology of nephrotic syndrome/FSGS; diagnosis may obviate the need for kidney biopsy; evaluation for structural urogenital abnormalities and screening for germ cell tumors; endocrine evaluation for hormonal abnormalities in individuals with disorders of testicular development	2
*OFD1*	*OFD1*-related conditions^48^ (orofaciodigital syndrome 1, joubert syndrome type 10, or Golabi–Behmel syndrome type 2)	Clarification of prognosis to atypical cystic disease Evaluation of orofacial malformations; hearing loss; skeletal abnormalities of hands and feet; dental abnormalities; growth delay; cardiac defects; structural brain abnormalities; liver abnormalities	2
*SALL1*	Townes–Brocks syndrome^49^	Evaluation of urinary tract abnormalities; cardiac abnormalities; ocular abnormalities; hearing loss	2
*PRKCSH*	Polycystic liver disease 1^50^	Clarification of prognosis to atypical cystic disease, diagnose liver cysts	1
*NPHS2*	Congenital nephrotic syndrome, type 2^51^	Diagnose the etiology of nephrotic syndrome/FSGS; may obviate the need for kidney biopsy	1
*TRPC6*	Focal segmental glomerulosclerosis 2^51^	Diagnose the etiology of nephrotic syndrome/FSGS; diagnosis may obviate the need for kidney biopsy	1
*ATP7B*	Wilson disease^52^	Evaluation for nephrolithiasis, liver disease, esophageal varices, movement disorders, mood disorders, and metabolic bone disorders	1
*DHCR7*	Smith–Lemli–Opitz syndrome^53^	Evaluation of gastrointestinal anomalies, liver disease, genitourinary abnormalities, neurologic disorders, eye disease/vision problems, hearing loss, growth deficiency, adrenal insufficiency, delayed puberty	1
*KANSL1*	Koolen–de vries syndrome^54^	Evaluation of structural kidney abnormalities; voiding abnormalities including vesicoureteral reflux; cryptorchidism; short stature/growth hormone replacement therapy; cardiac defects; seizures and structural brain abnormalities; hyperopia; strabismus; skeletal abnormalities including scoliosis, hip dislocation, and positional deformities of the feet	1
*LMNA*	Lipodystrophy type 2 and other disorders^55,56^	Evaluation structural and arrhythmogenic heart defects and hypertriglyceridemia; insulin resistance and diabetes; bone and vertebral anomalies; neuromuscular disease; pulmonary restriction; hypergonadotropic hypogonadism	1
*MAFB*	Multicentric carpotarsal osteolysis with or without nephropathy^57^	Evaluation of joint pain/inflammation; bone abnormalities; vision problems; hearing loss	1
*PAX2*	*PAX2*-related disorders (renal coloboma syndrome, focal segmental glomerulosclerosis 7; congenital abnormalities of the kidneys and urinary tract)^51^	Diagnose the etiology of nephrotic syndrome/FSGS, diagnosis may obviate the need for kidney biopsy, evaluate ocular abnormalities	1
*RRM2B*	RRM2B mitochondrial DNA maintenance defects^58^	Evaluation of encephalomyopathy, hypotonia, seizures, microcephaly, and developmental delay; hearing loss; progressive external ophthalmoplegia and other ocular abnormalities; cardiac abnormalities	1
*SMAD9*	Pulmonary hypertension, primary 2^59^	Cardiology/pulmonology for evaluation and management of pulmonary hypertension	1
*SMARCAL1*	Schimke immunoosseous dysplasia^60^	Evaluation of hematological abnormalities including anemia; T-cell deficiency and recurrent infections; ocular abnormalities including corneal opacity; hypodontia, microdontia, and other tooth abnormalities; neurovascular disease; vertebral anomalies, osteopenia and joint problems; hypothyroidism	1
*WAS*	Wiskott–Aldrich syndrome; X-linked thrombocytopenia; X-linked severe congenital neutropenia^61^	Evaluation for immune deficiency; evaluation for thrombocytopenia and neutropenia	1
Total			296

ADPKD, autosomal dominant polycystic kidney disease; AMKD, *APOL1*-mediated kidney disease; HANAC, hereditary angiopathy with nephropathy, aneurysms, and muscle cramps.

**Table 4:** Treatment and management implications of positive findings by gene

Gene(s)	Clinical Disease	Treatment and Management Implications	# Cases
*APOL1*	Susceptibility to kidney failure and FSGS	Multiple targeted therapies for patients with *APOL1* high-risk genotypes are expected soon. Preliminary results of ongoing clinical trials show efficacy in proteinuria reduction in *APOL1* high-risk patients	59
Atypical cystic genes *(ALG8, ALG9, IFT140, OFD1, PRKCSH)*	ADPKD-*ALG8*^43^; ADPKD-*ALG9*,^43^ ADPKD-*IFT140*^42^; *OFD1*-related conditions (orofaciodigital syndrome 1, Joubert syndrome type 10, or Golabi–Behmel syndrome type 2)^48^; polycystic liver disease 1^50^	Guides management given the likelihood of lower severity compared with *PKD1* or *PKD2*	17
*CASR*	*CASR*-related conditions (autosomal dominant hypocalcemia type 1 with or without Bartter syndrome; autosomal dominant familial hypocalciuric hypercalcemia type 1; autosomal recessive neonatal hyperparathyroidism)^62,63^	Causes FHH1 but overlaps with PHPT and differentiation is critical because of different treatment approaches	2
*CLCNKB*	Bartter syndrome, type 3/4B; Gitelman syndrome^64^	Diagnosis of Bartter syndrome type 3 distinguishes the clinical findings from Gitelman syndrome; treatment with sodium and potassium supplement (as opposed to Gitelman treatment of potassium sparing diuretics)	5
*COL4A1*	*COL4A1*-related disorders (HANAC; brain small vessel disease with hemorrhage)^65^	Avoid coagulant use and minimize high-risk behavior associated with hypertension, stroke, and head trauma	3
*COL4A3/4/5*	Alport syndrome^3^	Specific management recommendations, treatment with an inhibitor of the renin-angiotensin-aldosterone system, avoidance of immunosuppressive therapies, risk for kidney failure dependent on the *COL4A* gene (X-linked versus autosomal form of Alport syndrome)	61
*CUBN*	Chronic benign proteinuria^46^	Chronic proteinuria with retained kidney function guides management in most cases	2
*HNF1A*	*HNF1A*-maturity-onset diabetes of the young^66^	*HNF1A*-related monogenic diabetics can discontinue insulin and be effectively treated with sulfonylureas	4
Monogenic *FSGS*^51^ *NPHS2,*^67^ *PAX2, WT1*	Congenital nephrotic syndrome, type 2; *PAX2*-related disorders (renal coloboma syndrome, focal segmental glomerulosclerosis 7; congenital abnormalities of the kidneys and urinary tract)^68^; *WT1*-related disorders (Frasier syndrome; Denys–Drash syndrome; Meacham syndrome)^47^	Inform on use of immunosuppressive therapies	4
*PKD1*	ADPKD^32^	Inform on tolvaptan treatment	98
*PRKCSH*	Polycystic liver disease 1^50^	Management of liver cysts	1
*SLC12A3*	Gitelman syndrome^64^	Treatment with potassium-sparing diuretics, renin-angiotensin system inhibitors, daily oral potassium and magnesium supplements for life	5
*UMOD*	Autosomal dominant tubulointerstitial kidney disease^44,69^	Limited utility of biopsy, gout prophylaxis and low-purine diet. Re-evaluation of need for immunosuppression in rare patients presenting with FSGS^70^	4
Total			265

Patients with more than one gene finding (see Supplemental Table 5) were included in more than one row. ADPKD, autosomal dominant polycystic kidney disease; FHH1, familial hypocalciuric hypercalcemia type 1; HANAC, hereditary angiopathy with nephropathy, aneurysms, and muscle cramps; PHPT, primary hyperparathyroidism.

### Physician-Reported Changes in Management

**Figure 2:**
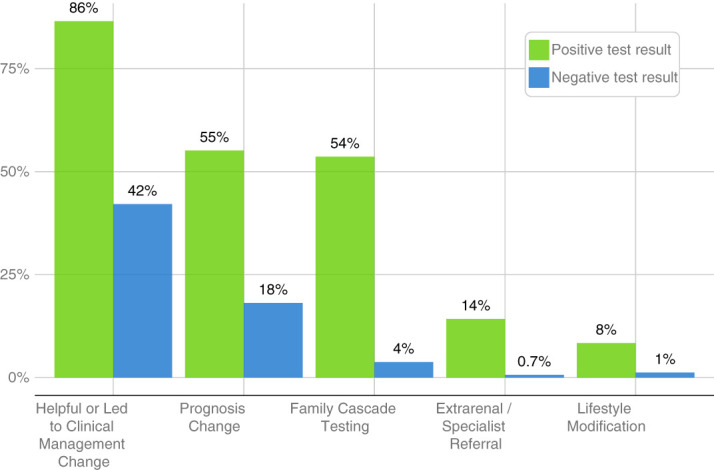
**Management and prognosis changes due to genetic testing results.** Family cascade testing, extrarenal specialist referral, and lifestyle modifications were the three most salient subcategories of management changes that were prompted by genetic testing results.

Overall, genetic testing influenced clinical management and/or led to changes in management in 86% (237/274) of patients with a positive test finding, and 42% (379/900, *P* < 0.001) of those with a negative test finding (Figure 2 and Supplemental Figure 1). Of the patients with a positive finding, 37% (102/274) experienced a single change in management, 12% (34/274) experienced two changes, and 6%, 4%, 1%, and 0.4% patients experienced three, four, five, and six changes, respectively; 21% (57/274) experienced a change in treatment, extrarenal referral, or additional workup, 16% (43/274) received other recommendations or referrals (*e.g*., to a clinical trial), and 54% (147/274) had family members referred for cascade testing or risk assessment; moreover, 83% (227/274) of physicians reported that the genetic testing was helpful for clinical management of these patients.

In addition, genetic testing was reported by physicians to result in a change in their expected 5-year prognosis for 27% (314/1174) of all patients, including 55% (151/274) of those with a positive test finding compared with 18% (163/900, *P* < 0.001) of those with a negative test finding (Supplemental Figure 2). Among those reporting a prognosis change, 51% (77/151) of patients with a positive gene finding had concern of either relapsing disease or rapid progression to kidney failure compared with 19% (31/163) of patients with a negative result.

Specific management changes resulting from positive gene findings were assessed in the questionnaire. Of the 274 patients with a positive test result, 54% (*n*=147) were recommended to pursue family cascade testing and follow-up risk assessments because of genetic test results. In addition, identification of extrarenal features or referral to a specialist was prominent among those with a positive genetic result, occurring in 14% (39/274) of cases. Furthermore, specific lifestyle changes based on genetic test results were more frequently recommended for patients with positive results compared with those with negative results (8% [23/274] versus 1% [11/900], *P* < 0.001; Figure 2 and Supplemental Table 4**)**.

### Exemplary Management Changes by Clinical Category

A detailed analysis of the six most common pretest clinical categories, accounting for 83% (279/335) of all positive cases, revealed diverse and clinically significant management changes following genetic testing. An extensive accounting of these examples is provided in the Supplemental Material.

### Diagnostic and Management Implications in Patients with Multiple Positive Gene Findings

In this study, 20 cases (6% of all positive cases) had more than one positive gene finding related to kidney disease (Supplemental Table 5). Half (10/20) of these cases with a dual or triple positive result had an *APOL1* high-risk genotype in addition to other positive gene findings. In three cases, (15%) the multiple positive findings were crucial for establishing a definitive etiology, particularly in cases with pathogenic variants in separate *COL4A3/4/5* genes, where similar disease presentations could have masked the accurate diagnosis. Overall, 85% (17/20) of these cases had findings that indicated disease processes with distinct clinical presentations. In addition, in 40% (8/20) of these cases, a combination of common and rare kidney-related diseases was detected within individual patients.

### Complementary Information with Kidney Biopsy and Positive Gene Findings

Among patients with a positive genetic test who also underwent a kidney biopsy, 69% (29/42), including 19 of 21 patients with FSGS, had histologic features that were consistent with their gene finding, thereby augmenting the diagnostic accuracy, and furthermore, suggesting that many biopsies could have been avoided with a genetics-first approach (Supplemental Table 6). Specific examples detailing the clinical utility of genetic testing in this cohort are available in the Supplemental Material.

### Transplant Implications Post-genetic Testing

One month following the issuance of test results, it was reported that a living related donor was under consideration for 42% (142/335) of patients with positive gene findings. At the 1-year post-test mark, questionnaire responses indicated a change in donor selection for 19% (15/80) of these patients, and the originally selected donor was not used. An additional two patients not considering a living related donor at 1 month indicated a transplant referral at 1 year involving a change in donor selection. These candidate transplant recipients had positive gene findings in *APOL1* (*n*=3), *HNF1A* (*n*=2), *PKD1* (*n*=2), *PKD2* (*n*=2), *TTR* (*n*=2), *CFH* (*n*=1), *CFI* (*n*=1), *COL4A3* (*n*=1), *COL4A4* (*n*=1), *NPHP1* (*n*=1), and *SLC4A1* (*n*=1).

### Currently Available Clinical Therapies and Clinical Trials

Currently, 24 therapeutics agents are available that target conditions caused by genetic variants that are covered by this 385-gene CKD panel (Supplemental Table 7). Consequently, 35% (120) of patients with a positive gene finding may be eligible for these existing therapies. In addition, there are approximately 112 ongoing US-based clinical trials related to CKD spanning phase 1–3 (Supplemental Table 8).

## Discussion

In this RenaCARE study analysis, including 1388 adults across 13 distinct pretest clinical categories of CKD, we demonstrated substantial diagnostic and clinical utility of broad-panel genetic testing after 1 year of prospective follow-up. According to 1174 physician questionnaire responses, genetic testing proved beneficial for clinical management or led to a management change in 86% of cases with a positive result and 42% of patients with a negative result. In addition, genetic testing altered the physician-reported 5-year prognosis for 55% of patients with positive findings and 18% of those with negative findings. Among the 335 patients with a positive gene finding, 96% had direct gene-based diagnostic, management, or treatment implications. These included establishing a definite diagnosis in patients with a nonspecific presentation, guiding assessments for both renal and extrarenal features, providing prognostic information including the likelihood of rapid disease progression, and influencing the use or discontinuation of available therapies, with 79% (265/335) having actionable treatment-related gene findings.

Importantly, both positive and negative gene findings have clinical utility for patients and their families. Negative gene findings provide utility by providing reassurance for patients and families, excluding the need for cascade testing, have impact on kidney donor considerations, and may provide guidance for further diagnostic workup or inform therapeutic decision making where the disease of genetic etiology requires different treatment, such as in FSGS.

The field of nephrology is embracing an era of personalized medicine, underscored by recent society recommendations endorsing genetic testing in the diagnosis and management of patients with CKD. The KDIGO 2024 Clinical Practice Guideline for the Evaluation and Management of Chronic Kidney Disease emphasizes the role of genetic testing in diagnostic evaluations, highlighting its potential to enhance patient self-care and early detection of familial disease.^10^ The inherent genetic and phenotypic heterogeneity in CKD means that a definitive genetic diagnosis can unify various clinical manifestations that are otherwise indistinguishable through conventional diagnostics. Genetic insights can clarify underlying pathophysiology and guide tailored treatments on the basis of precise diagnoses, and ultimately, early and accurate diagnoses can significantly improve CKD outcomes. This study uncovered myriad examples of clinical utility across the pretest clinical diagnosis cohorts, consonant with published guideline recommendations.

For example, in our study, patients with a pretest diagnosis of cystic nephropathy and positive gene findings showed typical and atypical presentations linked to different prognoses and treatment strategies, such as the use of tolvaptan. In addition, genetic testing facilitated family cascade testing and counseling about reproductive risks in over 90% of these patients, aligning with the KDIGO 2025 Clinical Practice Guideline for the Evaluation, Management, and Treatment of Autosomal Dominant Polycystic Kidney Disease (ADPKD) guidelines.^32^ These guidelines suggest that genetic testing can confirm the diagnosis, identify causal variants, and help to determine prognosis in all people in this category.^32^ A molecular diagnosis for those with cystic disease can be most beneficial by providing a clarifying prognosis that guides lifelong management, exemplified by cases with variants in genes associated with atypical cystic disease where 58.8% of responding nephrologists reported a change in prognosis to a slower or stable progression to disease because of the genetic result. Genetic testing was also beneficial in distinguishing patients with ADPKD from patients with other kidney disorders that may mimic ADPKD because of the common presence of kidney cysts. *OFD1* variants, found in two female patients, are associated with a syndromic ciliopathy that may present with cysts but can be differentiated from ADPKD by manifestations of oral, facial, and digital malformations and have a worse prognosis in male patients. Other positive noncystic gene findings in this category (*SLC7A9*, *TRPC6*) have distinct clinical features with defined therapeutic options, making genetic testing crucial to optimize the outcomes in these patients. Given that cyst development is a common manifestation in kidney disease, the ADPKD guidelines emphasize the importance of being able to differentiate the forms of heritable cystic kidney disease through genetic testing.^32^

The utility of genetic testing is also highlighted in conditions like Alport syndrome (also referred to as Alport spectrum or *COL4A*-related nephropathy), where studies have shown that up to 62% of patients are misdiagnosed.^12^ Among patients with proteinuria due to glomerular CKD, positive gene findings had a significant impact on management changes (89.2%), evaluation for extrarenal features (13.5%), family cascade testing (51.4%), and provided clarity on disease progression (51.4%). Importantly, the identification of a genetic etiology in patients with glomerulopathy enabled the timely treatment of patients with *COL4A*-related diseases with renin-angiotensin-aldosterone system (RAAS) blockade or sodium-glucose cotransporter 2 inhibitors, which can slow the decline of eGFR and disease progression and, ultimately, improve outcomes. Specifically, genetic testing can clarify the variant type, location, and mode of inheritance for the *COL4A3/4/5* variants (autosomal dominant, autosomal recessive, or X-linked Alport syndrome), which affects the risk of progression to kidney failure and informs initiation of appropriate therapies and could guide referral to clinical trials. This is consistent with the current guideline recommendations to identify all individuals with pathogenic heterozygous *COL4A3/4* variants, or women with *COL4A5* variant, as early as possible, and treat them with RAAS blockade from the onset of microalbuminuria, hypertension, or kidney impairment, while men with a *COL4A5* variant should be treated with RAAS blockade at the time of diagnosis.^3^

Some patients with a pretest clinical diagnosis of nephropathy associated with hypertension or diabetes mellitus had gene-based diagnoses confirming that their CKD was unrelated to these conditions, necessitating tailored management. For instance, the detection of a homozygous *NPHP1* gene deletion in a hypertensive patient, indicative of autosomal recessive nephronophthisis, led to an ophthalmological assessment for retinal dystrophy and additional radiologic evaluations for kidney and liver abnormalities, consistent with published guidance.^33^ This molecular diagnosis eliminated the need for a kidney biopsy. Similarly, a finding of *COL4A1* necessitated evaluations for hereditary angiopathy with nephropathy, aneurysms, and muscle cramps, with management adjustments including early referral to cardiology to prevent arrhythmias and avoidance of anticoagulants to mitigate stroke risk. Furthermore, some patients initially diagnosed with diabetes-related nephropathy were found to have *HNF1A*-MODY, benefiting from low-dose sulfonylureas instead of insulin. These gene findings altered medical management, adjusting prognosis expectations and expediting transplant referrals.

Diagnoses in patients with kidney disease due to unknown etiology often remain elusive because of phenotypic heterogeneity in patients with CKD. Previous studies indicate about 20% of these patients harbor genetic variants implicated in CKD, reflected by a 19.1% yield rate in our cohort.^34^ Positive gene findings linked to unique syndromes like Townes–Brocks syndrome (*SALL1*), Bartter syndrome (*CLCNKB*), and *WT1*-associated disorders often require specialized management plans. NKF recommendations underscore the importance of genetic testing in this group to ensure accurate diagnoses.^9^

Patients with multiple gene findings present complex diagnostic challenges. In the RenaCARE cohort, 20 patients (6% of positive cases) had multiple genetic variants, complicating their clinical presentations. Identifying these conditions often resolves disparate symptoms and facilitates comprehensive management strategies, as seen in cases with *PKD2*/*SLC4A1*, *COL4A3*/*SLC12A3*, and *CASR*/*SALL1*, which require both electrolyte management and specialist referrals.

In the kidney failure cohort, genetic testing influenced clinical management in 87% of cases with positive findings. A recent study has demonstrated that patients with genetic forms of CKD progress faster, yet these patients may have better survival after they reach kidney failure.^35,36^ Therefore, as these patients progress toward KRT or transplantation, genetic insights provide a more precise prognosis and can optimize referral timing. For example, patients with primary FSGS have a high post-transplant recurrence risk,^37^ whereas patients with genetic FSGS linked to variants like *APOL1*, *COL4A3*, *COL4A4*, and *MAFB* typically show low recurrence rates.^38^ One patient with a *CFH* variant was identified as having a high risk of atypical hemolytic uremic syndrome, requiring targeted post-transplant therapy to avoid graft failure.^39,40^

The selection of living donors for transplantation has evolved, especially for candidates with identified genetic risks. The American Society of Transplantation advises genetic testing for potential donors related to candidates with known genetic kidney diseases and recommends against donation from those with certain *COL4A3/4/5* variants because of their own risk of kidney impairment.^3^ In this cohort, 17 patients had a positive gene finding that ultimately resulted in the selection of another kidney donor because of these additional risks.

Genetic testing complements traditional diagnostic approaches like biopsies, enhancing management by providing detailed genetic information that can influence treatment choices. For instance, in patients with FSGS detected on biopsy, identifying specific gene variants (*COL4A3/4/5*, *APOL1*, *NPHS2*) can guide decisions regarding immunosuppression. In addition, nonspecific findings on biopsy can be clarified by certain positive gene findings including *CUBN* (chronic proteinuria) and *UMOD* (ADTKD). Recognizing the underlying kidney disease can assist in the management of these patients.

The primary limitation of this study is that our findings reflect clinician-reported perspectives rather than observed clinical outcomes. As such, the results represent perceived utility and anticipated or intended changes in care. Nonetheless, we believe that these data provide critical insights into the ways nephrologists integrate genetic information into practice. Some questions in the questionnaire may not have fully captured the motivations behind management decisions or some of the specific aspects of those decisions. Some data omissions may have resulted from situations where questions addressed issues that were not within the clinician's direct scope of care. Furthermore, because the cystic nephropathies, nephropathies associated with hypertension, and nephropathies associated with diabetes mellitus cohorts were capped, they may be underrepresented in this cohort compared with a clinical CKD population, and consequently, other less common CKD subgroups may be overrepresented in this cohort. Similarly, we cannot rule out disproportionate representation in this cohort because of preferential enrollment by physicians, or differing clinical diagnoses for patients with similar clinical features. Another limitation of this study is that it was conducted exclusively in the United States, and as such, the study population reflects the demographic makeup of the regions served by participating clinical sites. Consequently, the findings may not be generalizable to global populations or to ethnic groups that were underrepresented in this cohort. Given that the prevalence, pathogenicity, and clinical interpretation of gene variants can vary significantly across ancestries, these results should be considered primarily applicable to the US population. Although this study was designed with a defined scope, it has illuminated several important avenues for future research. Key questions remain around the management of incidental findings, the interplay between perceived utility and clinical outcomes, and factors influencing donor selection. Addressing these issues in subsequent studies will be critical to advancing understanding and maximizing the clinical and translational impact of this field. Finally, several relevant genetic testing guidelines were not available during the study, possibly limiting the nephrologists' interpretation skills and subsequent management adjustments. This highlights the ongoing need for enhanced education about genetic testing in CKD management.

In conclusion, the RenaCARE study affirmed the clinical utility of genetic testing in CKD management as reported by physicians and further illustrated the high aggregate prevalence of rare diseases in nephrology practice, supporting the broader use of genetic testing in nephrology. This approach enhances understanding of the genetic underpinnings of CKD and fosters the development of targeted therapies, advancing a new era in nephrology that will fundamentally transform clinical practice. The results offer insights into physicians' perspective regarding the utility of genetic testing for CKD and also highlight opportunities for further education around the potential benefits of genetic testing for both physicians and patients.

## Supplementary Material

## Data Availability

Original data generated for the study will be made available upon reasonable request to the corresponding author. Data Type: Aggregated Data. Reason for Restricted Access: To protect patient privacy.

## Declarative Statements

This study includes clinical experimentation and received Institutional Review Board or Ethics Committee approval. All patients provided written informed consent. This study includes clinical experimentation and complies with the Declaration of Helsinki. The clinical and research activities being reported are consistent with the Principles of the Declaration of Istanbul as outlined in the Declaration of Istanbul on Organ Trafficking and Transplant Tourism.

## Supplemental Material

Supplemental Table 1. Study inclusion and exclusion criteria.

Supplemental Table 2. Physician 1-year Post-test Questionnaire.

Supplemental Table 3. Demographics for the full 1388-patient cohort.

Supplemental Table 4. Management and prognosis changes based on genetic test results.

Supplemental Table 5. Genetic findings in patients with multiple genetic findings.

Supplemental Table 6. Pretest and post-test diagnoses for 29 patients with a complementary kidney biopsy.

Supplemental Table 7. US Food and Drug Administration–approved treatments for patients with conditions covered by the Renasight panel.

Supplemental Table 8. US phase 1–3 trials for target CKD conditions.

Supplemental Figure 1. Proportion of patients with positive or negative test findings whose genetic testing influenced clinical management and/or led to changes in management, stratified by clinical diagnosis.

Supplemental Figure 2. Proportion of patients with positive or negative test findings whose physician reported that genetic testing led to a change in their expected 5-year prognosis, stratified by clinical diagnosis.
