# Supplementary material for: Genetic Testing in the Management of Adult CKD
Source: J Am Soc Nephrol. 2025 Oct 29;37(4):777–89. doi: 10.1681/ASN.0000000913 (PMC13065169; doi:10.1681/ASN.0000000913)
Supplement: Supplementary file 1 [file jasn-37-777-s001.pdf]

ASN American Society of Nephrology | JASN Journal of the American Society of Nephrology | KIDNEY360 Accessing Our World From Every Angle | CJASN Clinical Journal of the American Society of Nephrology

ASN Journal Disclosure Form

As per ASN journal policy, I have disclosed any financial relationships or commitments I have held in the past 36 months as included below. I have listed my Current Employer below to indicate there is a relationship requiring disclosure. If no relationship exists, my Current Employer is not listed.

S. Bhorade reports the following:
Employer: Natera; Ownership Interest: Natera; Veracyte; and Patents or Royalties: Natera.

I understand that the information above will be published within the journal article, if accepted, and that failure to comply and/or to accurately and completely report the potential financial conflicts of interest could lead to the following: 1) Prior to publication, article rejection, or 2) Post-publication, sanctions ranging from, but not limited to, issuing a correction, reporting the inaccurate information to the authors' institution, banning authors from submitting work to ASN journals for varying lengths of time, and/or retraction of the published work.

Name: Sangeeta Bhorade
Manuscript ID: JASN-2025-000496R1
Manuscript Title: Clinical Utility of Genetic Testing in Adults with Chronic Kidney Disease
Date of Completion: August 21, 2025
Disclosure Updated Date: August 21, 2025

ASN American Society of Nephrology
JASN Journal of the American Society of Nephrology
KIDNEY360 Accessing Our World From Every Angle
CJASN Clinical Journal of the American Society of Nephrology

ASN Journal Disclosure Form

As per ASN journal policy, I have disclosed any financial relationships or commitments I have held in the past 36 months as included below. I have listed my Current Employer below to indicate there is a relationship requiring disclosure. If no relationship exists, my Current Employer is not listed.

M. Bloom reports the following:
Employer: Natera, Inc.; and Ownership Interest: Natera, Inc.

I understand that the information above will be published within the journal article, if accepted, and that failure to comply and/or to accurately and completely report the potential financial conflicts of interest could lead to the following: 1) Prior to publication, article rejection, or 2) Post-publication, sanctions ranging from, but not limited to, issuing a correction, reporting the inaccurate information to the authors' institution, banning authors from submitting work to ASN journals for varying lengths of time, and/or retraction of the published work.

Name: Michelle Bloom
Manuscript ID: JASN-2025-000496R1
Manuscript Title: Clinical Utility of Genetic Testing in Adults with Chronic Kidney Disease
Date of Completion: August 20, 2025
Disclosure Updated Date: August 20, 2025

ASN American Society of Nephrology
JASN Journal of the American Society of Nephrology
KIDNEY360 Accessing Our World From Every Angle
CJASN Clinical Journal of the American Society of Nephrology

ASN Journal Disclosure Form

As per ASN journal policy, I have disclosed any financial relationships or commitments I have held in the past 36 months as included below. I have listed my Current Employer below to indicate there is a relationship requiring disclosure. If no relationship exists, my Current Employer is not listed.

S. Chapman reports the following:
Employer: Natera, Inc; Ownership Interest: Natera, Inc; and Advisory or Leadership Role: Board Member for ACLA.

I understand that the information above will be published within the journal article, if accepted, and that failure to comply and/or to accurately and completely report the potential financial conflicts of interest could lead to the following: 1) Prior to publication, article rejection, or 2) Post-publication, sanctions ranging from, but not limited to, issuing a correction, reporting the inaccurate information to the authors' institution, banning authors from submitting work to ASN journals for varying lengths of time, and/or retraction of the published work.

Name: Steve Chapman
Manuscript ID: JASN-2025-000496R1
Manuscript Title: Clinical Utility of Genetic Testing in Adults with Chronic Kidney Disease
Date of Completion: August 13, 2025
Disclosure Updated Date: August 13, 2025

ASN
American Society of Nephrology
JASN
Journal of the American Society of Nephrology
KIDNEY360
Accessing Our World From Every Angle
CJASN
Clinical Journal of the American Society of Nephrology

ASN Journal Disclosure Form

As per ASN journal policy, I have disclosed any financial relationships or commitments I have held in the past 36 months as included below. I have listed my Current Employer below to indicate there is a relationship requiring disclosure. If no relationship exists, my Current Employer is not listed.

F. Chebib reports the following:
Employer: Mayo Clinic; Research Funding: Research grant- Otsuka pharmaceuticals; Natera; Regulus; Vertex; and Patents or Royalties: Patent no US20200368191A1.

I understand that the information above will be published within the journal article, if accepted, and that failure to comply and/or to accurately and completely report the potential financial conflicts of interest could lead to the following: 1) Prior to publication, article rejection, or 2) Post-publication, sanctions ranging from, but not limited to, issuing a correction, reporting the inaccurate information to the authors' institution, banning authors from submitting work to ASN journals for varying lengths of time, and/or retraction of the published work.

Name: Fouad T. Chebib
Manuscript ID: JASN-2025-000496R1
Manuscript Title: Clinical Utility of Genetic Testing in Adults with Chronic Kidney Disease
Date of Completion: July 31, 2025
Disclosure Updated Date: May 8, 2025

ASN American Society of Nephrology | JASN Journal of the American Society of Nephrology | KIDNEY360 Accessing Our World From Every Angle | CJASN Clinical Journal of the American Society of Nephrology

ASN Journal Disclosure Form

As per ASN journal policy, I have disclosed any financial relationships or commitments I have held in the past 36 months as included below. I have listed my Current Employer below to indicate there is a relationship requiring disclosure. If no relationship exists, my Current Employer is not listed.

D. Clark reports the following:
Employer: Natera; and Ownership Interest: Natera.

I understand that the information above will be published within the journal article, if accepted, and that failure to comply and/or to accurately and completely report the potential financial conflicts of interest could lead to the following: 1) Prior to publication, article rejection, or 2) Post-publication, sanctions ranging from, but not limited to, issuing a correction, reporting the inaccurate information to the authors' institution, banning authors from submitting work to ASN journals for varying lengths of time, and/or retraction of the published work.

Name: Dinah Clark
Manuscript ID: JASN-2025-000496R1
Manuscript Title: Clinical Utility of Genetic Testing in Adults with Chronic Kidney Disease
Date of Completion: August 20, 2025
Disclosure Updated Date: August 20, 2025

ASN
American Society of Nephrology
JASN
Journal of the American Society of Nephrology
KIDNEY360
Accessing Our World From Every Angle
CJASN
Clinical Journal of the American Society of Nephrology

ASN Journal Disclosure Form

As per ASN journal policy, I have disclosed any financial relationships or commitments I have held in the past 36 months as included below. I have listed my Current Employer below to indicate there is a relationship requiring disclosure. If no relationship exists, my Current Employer is not listed.

N. Dahl reports the following:
Employer: Mayo Clinic; Consultancy: Renasant Bio, Vertex, Regulus, Estuary Bio; Research Funding: I am a PI for clinical trials sponsored by Vertex and AbbVie.; Honoraria: Natera; Advisory or Leadership Role: Natera Scientific Advisory Board; and Other Interests or Relationships: Associate Editor, Kidney360, Scientific Advisory Board, PKD Foundation.

I understand that the information above will be published within the journal article, if accepted, and that failure to comply and/or to accurately and completely report the potential financial conflicts of interest could lead to the following: 1) Prior to publication, article rejection, or 2) Post-publication, sanctions ranging from, but not limited to, issuing a correction, reporting the inaccurate information to the authors' institution, banning authors from submitting work to ASN journals for varying lengths of time, and/or retraction of the published work.

Name: Neera K. Dahl
Manuscript ID: JASN-2025-000496R1
Manuscript Title: Clinical Utility of Genetic Testing in Adults with Chronic Kidney Disease
Date of Completion: August 13, 2025
Disclosure Updated Date: August 13, 2025

ASN American Society of Nephrology · JASN Journal of the American Society of Nephrology · KIDNEY360 Accessing Our World From Every Angle · CJASN Clinical Journal of the American Society of Nephrology

ASN Journal Disclosure Form

As per ASN journal policy, I have disclosed any financial relationships or commitments I have held in the past 36 months as included below. I have listed my Current Employer below to indicate there is a relationship requiring disclosure. If no relationship exists, my Current Employer is not listed.

Z. Demko reports the following:
Employer: Natera; and Ownership Interest: I own stock in Natera.

I understand that the information above will be published within the journal article, if accepted, and that failure to comply and/or to accurately and completely report the potential financial conflicts of interest could lead to the following: 1) Prior to publication, article rejection, or 2) Post-publication, sanctions ranging from, but not limited to, issuing a correction, reporting the inaccurate information to the authors' institution, banning authors from submitting work to ASN journals for varying lengths of time, and/or retraction of the published work.

Name: Zachary Demko
Manuscript ID: JASN-2025-000496R1
Manuscript Title: Clinical Utility of Genetic Testing in Adults with Chronic Kidney Disease
Date of Completion: August 14, 2025
Disclosure Updated Date: August 14, 2025

ASN
American Society of Nephrology
JASN
Journal of the American Society of Nephrology
KIDNEY360
Accessing Our World From Every Angle
CJASN
Clinical Journal of the American Society of Nephrology

ASN Journal Disclosure Form

As per ASN journal policy, I have disclosed any financial relationships or commitments I have held in the past 36 months as included below. I have listed my Current Employer below to indicate there is a relationship requiring disclosure. If no relationship exists, my Current Employer is not listed.

N. Dossabhoy reports the following:
Employer: Univ. of Mississippi Medical Center; Ownership Interest: I own diversified mutual funds, ETFs, and/or stock options in Tesla; ATT; Verizon; T-Mobile; Uber; etc.; and Other Interests or Relationships: I serve on the CME committee for the National Kidney Foundation.

I understand that the information above will be published within the journal article, if accepted, and that failure to comply and/or to accurately and completely report the potential financial conflicts of interest could lead to the following: 1) Prior to publication, article rejection, or 2) Post-publication, sanctions ranging from, but not limited to, issuing a correction, reporting the inaccurate information to the authors' institution, banning authors from submitting work to ASN journals for varying lengths of time, and/or retraction of the published work.

Name: Neville R. Dossabhoy
Manuscript ID: JASN-2025-000496R1
Manuscript Title: Clinical Utility of Genetic Testing in Adults with Chronic Kidney Disease
Date of Completion: August 28, 2025
Disclosure Updated Date: August 28, 2025

ASN
American Society of Nephrology
JASN
Journal of the American Society of Nephrology
KIDNEY360
Accessing Our World From Every Angle
CJASN
Clinical Journal of the American Society of Nephrology

ASN Journal Disclosure Form

As per ASN journal policy, I have disclosed any financial relationships or commitments I have held in the past 36 months as included below. I have listed my Current Employer below to indicate there is a relationship requiring disclosure. If no relationship exists, my Current Employer is not listed.

A. Faravardeh reports the following:
Employer: SHARP Kidney and Pancreas Transplant Center; and Advisory or Leadership Role: CareDx, Natera.

I understand that the information above will be published within the journal article, if accepted, and that failure to comply and/or to accurately and completely report the potential financial conflicts of interest could lead to the following: 1) Prior to publication, article rejection, or 2) Post-publication, sanctions ranging from, but not limited to, issuing a correction, reporting the inaccurate information to the authors' institution, banning authors from submitting work to ASN journals for varying lengths of time, and/or retraction of the published work.

Name: Arman Faravardeh
Manuscript ID: JASN-2025-000496R1
Manuscript Title: “Clinical Utility of Genetic Testing in Adults with Chronic Kidney Disease.”
Date of Completion: August 28, 2025
Disclosure Updated Date: August 28, 2025

ASN American Society of Nephrology
JASN Journal of the American Society of Nephrology
KIDNEY360 Accessing Our World From Every Angle
CJASN Clinical Journal of the American Society of Nephrology

ASN Journal Disclosure Form

As per ASN journal policy, I have disclosed any financial relationships or commitments I have held in the past 36 months as included below. I have listed my Current Employer below to indicate there is a relationship requiring disclosure. If no relationship exists, my Current Employer is not listed.

A. Gharavi reports the following:
Employer: Columbia University; Consultancy: Natera;Actio biosciences, Novartis: Travere; Calliditas;Vera, Vertex; Research Funding: Natera; and Advisory or Leadership Role: Editorial board: Science Advances.

I understand that the information above will be published within the journal article, if accepted, and that failure to comply and/or to accurately and completely report the potential financial conflicts of interest could lead to the following: 1) Prior to publication, article rejection, or 2) Post-publication, sanctions ranging from, but not limited to, issuing a correction, reporting the inaccurate information to the authors' institution, banning authors from submitting work to ASN journals for varying lengths of time, and/or retraction of the published work.

Name: Ali G. Gharavi
Manuscript ID: JASN-2025-000496R1
Manuscript Title: Clinical Utility of Genetic Testing in Adults with Chronic Kidney Disease
Date of Completion: August 14, 2025
Disclosure Updated Date: April 7, 2025

ASN American Society of Nephrology
JASN Journal of the American Society of Nephrology
KIDNEY360 Accessing Our World From Every Angle
CJASN Clinical Journal of the American Society of Nephrology

ASN Journal Disclosure Form

As per ASN journal policy, I have disclosed any financial relationships or commitments I have held in the past 36 months as included below. I have listed my Current Employer below to indicate there is a relationship requiring disclosure. If no relationship exists, my Current Employer is not listed.

V. Kolupaeva reports the following:
Employer: Columbia University Medical Center

I understand that the information above will be published within the journal article, if accepted, and that failure to comply and/or to accurately and completely report the potential financial conflicts of interest could lead to the following: 1) Prior to publication, article rejection, or 2) Post-publication, sanctions ranging from, but not limited to, issuing a correction, reporting the inaccurate information to the authors' institution, banning authors from submitting work to ASN journals for varying lengths of time, and/or retraction of the published work.

Name: Victoria Kolupaeva
Manuscript ID: JASN-2025-000496R1
Manuscript Title: Clinical Utility of Genetic Testing in Adults with Chronic Kidney Disease.
Date of Completion: August 26, 2025
Disclosure Updated Date: August 13, 2025

ASN Journal Disclosure Form

As per ASN journal policy, I have disclosed any financial relationships or commitments I have held in the past 36 months as included below. I have listed my Current Employer below to indicate there is a relationship requiring disclosure. If no relationship exists, my Current Employer is not listed.

S. Kotte reports the following:
Employer: Natera; Consultancy: Alderbrook; and Ownership Interest: Natera, Amazon, Nvidia & Apple.

I understand that the information above will be published within the journal article, if accepted, and that failure to comply and/or to accurately and completely report the potential financial conflicts of interest could lead to the following: 1) Prior to publication, article rejection, or 2) Post-publication, sanctions ranging from, but not limited to, issuing a correction, reporting the inaccurate information to the authors' institution, banning authors from submitting work to ASN journals for varying lengths of time, and/or retraction of the published work.

Name: Srinath Kotte
Manuscript ID: JASN-2025-000496R1
Manuscript Title: Clinical Utility of Genetic Testing in Adults with Chronic Kidney Disease
Date of Completion: August 20, 2025
Disclosure Updated Date: August 20, 2025

ASN American Society of Nephrology JASN Journal of the American Society of Nephrology KIDNEY360 Accessing Our World From Every Angle CJASN Clinical Journal of the American Society of Nephrology

ASN Journal Disclosure Form

As per ASN journal policy, I have disclosed any financial relationships or commitments I have held in the past 36 months as included below. I have listed my Current Employer below to indicate there is a relationship requiring disclosure. If no relationship exists, my Current Employer is not listed.

H. Milo Rasouly reports the following:
Employer: Columbia University; and Research Funding: Natera.

I understand that the information above will be published within the journal article, if accepted, and that failure to comply and/or to accurately and completely report the potential financial conflicts of interest could lead to the following: 1) Prior to publication, article rejection, or 2) Post-publication, sanctions ranging from, but not limited to, issuing a correction, reporting the inaccurate information to the authors' institution, banning authors from submitting work to ASN journals for varying lengths of time, and/or retraction of the published work.

Name: Hila Milo Rasouly
Manuscript ID: JASN-2025-000496R1
Manuscript Title: Clinical Utility of Genetic Testing in Adults with Chronic Kidney Disease
Date of Completion: August 21, 2025
Disclosure Updated Date: March 27, 2025

ASN American Society of Nephrology | JASN Journal of the American Society of Nephrology | KIDNEY360 Accessing Our World From Every Angle | CJASN Clinical Journal of the American Society of Nephrology

ASN Journal Disclosure Form

As per ASN journal policy, I have disclosed any financial relationships or commitments I have held in the past 36 months as included below. I have listed my Current Employer below to indicate there is a relationship requiring disclosure. If no relationship exists, my Current Employer is not listed.

M. Mizani reports the following:
Employer: South Texas Renal Care Group; and Research Funding: Natera.

I understand that the information above will be published within the journal article, if accepted, and that failure to comply and/or to accurately and completely report the potential financial conflicts of interest could lead to the following: 1) Prior to publication, article rejection, or 2) Post-publication, sanctions ranging from, but not limited to, issuing a correction, reporting the inaccurate information to the authors' institution, banning authors from submitting work to ASN journals for varying lengths of time, and/or retraction of the published work.

Name: Mohammad R. Mizani
Manuscript ID: JASN-2025-000496R1
Manuscript Title: Clinical Utility of Genetic Testing in Adults with Chronic Kidney Disease
Date of Completion: August 28, 2025
Disclosure Updated Date: August 28, 2025

ASN American Society of Nephrology JASN Journal of the American Society of Nephrology KIDNEY360 Accessing Our World From Every Angle CJASN Clinical Journal of the American Society of Nephrology

ASN Journal Disclosure Form

As per ASN journal policy, I have disclosed any financial relationships or commitments I have held in the past 36 months as included below. I have listed my Current Employer below to indicate there is a relationship requiring disclosure. If no relationship exists, my Current Employer is not listed.

S. Punj reports the following:
Employer: Natera Inc; and Ownership Interest: Natera Inc.

I understand that the information above will be published within the journal article, if accepted, and that failure to comply and/or to accurately and completely report the potential financial conflicts of interest could lead to the following: 1) Prior to publication, article rejection, or 2) Post-publication, sanctions ranging from, but not limited to, issuing a correction, reporting the inaccurate information to the authors' institution, banning authors from submitting work to ASN journals for varying lengths of time, and/or retraction of the published work.

Name: Sumit Punj
Manuscript ID: JASN-2025-000496R1
Manuscript Title: Clinical Utility of Genetic Testing in Adults with Chronic Kidney Disease
Date of Completion: August 13, 2025
Disclosure Updated Date: August 13, 2025

ASN American Society of Nephrology | JASN Journal of the American Society of Nephrology | KIDNEY360 Accessing Our World From Every Angle | CJASN Clinical Journal of the American Society of Nephrology

ASN Journal Disclosure Form

As per ASN journal policy, I have disclosed any financial relationships or commitments I have held in the past 36 months as included below. I have listed my Current Employer below to indicate there is a relationship requiring disclosure. If no relationship exists, my Current Employer is not listed.

M. Rabinowitz reports the following:
Employer: Natera Inc.; MyOme ; Centinus (FKA NatureEye) ; Medici Therapeutics (Marble Therapeutics); 1AU; Ownership Interest: MyOme ; Natera; Medici Therapeutics (FKA Marble Therapeutics); Research Funding: Yes, I've been involved in research funding for companies Natera, Myome, and Medici Therapeutics; Patents or Royalties: Natera, Myome, and Medici Therapeutics (FKA Marble Therapeutics); Advisory or Leadership Role: Natera Inc (Paid).; MyOme (paid) ; Centinus (FKA NatureEye) ; Medici Therapeutics (FKA Marble Therapeutics (paid) ; 1AU (paid); and Other Interests or Relationships: I served on the board of the American College of Medical Genetics Foundation.

I understand that the information above will be published within the journal article, if accepted, and that failure to comply and/or to accurately and completely report the potential financial conflicts of interest could lead to the following: 1) Prior to publication, article rejection, or 2) Post-publication, sanctions ranging from, but not limited to, issuing a correction, reporting the inaccurate information to the authors' institution, banning authors from submitting work to ASN journals for varying lengths of time, and/or retraction of the published work.

Name: Matthew Rabinowitz
Manuscript ID: JASN-2025-000496R1
Manuscript Title: Title: Clinical Utility of Genetic Testing in Adults with Chronic Kidney Disease
Date of Completion: September 2, 2025
Disclosure Updated Date: September 2, 2025

ASN
American Society of Nephrology
JASN
Journal of the American Society of Nephrology
KIDNEY360
Accessing Our World From Every Angle
CJASN
Clinical Journal of the American Society of Nephrology

ASN Journal Disclosure Form

As per ASN journal policy, I have disclosed any financial relationships or commitments I have held in the past 36 months as included below. I have listed my Current Employer below to indicate there is a relationship requiring disclosure. If no relationship exists, my Current Employer is not listed.

R. Schneider reports the following:
Employer: Natera;; Ownership Interest: Natera; Research Funding: Natera; Patents or Royalties: Boston Children's Hospital; and Advisory or Leadership Role: Vortex-Imaging.

I understand that the information above will be published within the journal article, if accepted, and that failure to comply and/or to accurately and completely report the potential financial conflicts of interest could lead to the following: 1) Prior to publication, article rejection, or 2) Post-publication, sanctions ranging from, but not limited to, issuing a correction, reporting the inaccurate information to the authors’ institution, banning authors from submitting work to ASN journals for varying lengths of time, and/or retraction of the published work.

Name: Ronen Schneider
Manuscript ID: JASN-2025-000496R1
Manuscript Title: Clinical Utility of Genetic Testing in Adults with Chronic Kidney Disease
Date of Completion: July 31, 2025
Disclosure Updated Date: July 31, 2025

ASN American Society of Nephrology
JASN Journal of the American Society of Nephrology
KIDNEY360 Accessing Our World From Every Angle
CJASN Clinical Journal of the American Society of Nephrology

ASN Journal Disclosure Form

As per ASN journal policy, I have disclosed any financial relationships or commitments I have held in the past 36 months as included below. I have listed my Current Employer below to indicate there is a relationship requiring disclosure. If no relationship exists, my Current Employer is not listed.

H. Tabriziani reports the following:
Employer: I am full time employee of Natera as the senior medical director of organ health and transplantation; Consultancy: Natera; Ownership Interest: Natera; Honoraria: Natera; Advisory or Leadership Role: Natera; Speakers Bureau: Natera; and Other Interests or Relationships: HossMed, Inc.

I understand that the information above will be published within the journal article, if accepted, and that failure to comply and/or to accurately and completely report the potential financial conflicts of interest could lead to the following: 1) Prior to publication, article rejection, or 2) Post-publication, sanctions ranging from, but not limited to, issuing a correction, reporting the inaccurate information to the authors' institution, banning authors from submitting work to ASN journals for varying lengths of time, and/or retraction of the published work.

Name: Hossein Tabriziani
Manuscript ID: JASN-2025-000496R2
Manuscript Title: Impact of Genetic Testing on Adult Chronic Kidney Disease (CKD) Management: Reported Utility After One Year.
Date of Completion: September 29, 2025
Disclosure Updated Date: September 29, 2025

ASN American Society of Nephrology | JASN Journal of the American Society of Nephrology | KIDNEY360 Accessing Our World From Every Angle | CJASN Clinical Journal of the American Society of Nephrology

ASN Journal Disclosure Form

As per ASN journal policy, I have disclosed any financial relationships or commitments I have held in the past 36 months as included below. I have listed my Current Employer below to indicate there is a relationship requiring disclosure. If no relationship exists, my Current Employer is not listed.

S. Udani reports the following:
Employer: Nephrology Assocaties of Northern Illinois; Consultancy: Natera; Abbott; Boehringer-Ingelheim, Travere, Calliditas, Amgen, George Clinical Institute, Novartis, Apellis, SC Pharmaceuticals; Ownership Interest: Strive Health; Research Funding: Travere; Novartis; Astra-Zeneca; Dimerix; Bayer; Sanofi; Walden Bioscience; Enyo, Apellis; Honoraria: Boehringer-Ingelheim, Travere, Novartis, Calliditas, Amgen, Apellis; Advisory or Leadership Role: Nephcure Kidney Network; National Kidney Foundation of Illinois; Speakers Bureau: Boehringer-Ingelheim; Travere, Amgen, Novartis, Apellis, SC Pharmaceuticals; and Other Interests or Relationships: National Kidney Foundation; Nephcure.

I understand that the information above will be published within the journal article, if accepted, and that failure to comply and/or to accurately and completely report the potential financial conflicts of interest could lead to the following: 1) Prior to publication, article rejection, or 2) Post-publication, sanctions ranging from, but not limited to, issuing a correction, reporting the inaccurate information to the authors' institution, banning authors from submitting work to ASN journals for varying lengths of time, and/or retraction of the published work.

Name: Suneel M. Udani
Manuscript ID: JASN-2025-000496R1
Manuscript Title: Clinical Utility of Genetic Testing in Adults with Chronic Kidney Disease
Date of Completion: August 28, 2025
Disclosure Updated Date: August 28, 2025

ASN American Society of Nephrology
JASN Journal of the American Society of Nephrology
KIDNEY360 Accessing Our World From Every Angle
CJASN Clinical Journal of the American Society of Nephrology

ASN Journal Disclosure Form

As per ASN journal policy, I have disclosed any financial relationships or commitments I have held in the past 36 months as included below. I have listed my Current Employer below to indicate there is a relationship requiring disclosure. If no relationship exists, my Current Employer is not listed.

X. Wang reports the following:
Employer: Cleveland Clinic; Consultancy: Natera; and Other Interests or Relationships: Member of ASN, NKF and ISN.

I understand that the information above will be published within the journal article, if accepted, and that failure to comply and/or to accurately and completely report the potential financial conflicts of interest could lead to the following: 1) Prior to publication, article rejection, or 2) Post-publication, sanctions ranging from, but not limited to, issuing a correction, reporting the inaccurate information to the authors' institution, banning authors from submitting work to ASN journals for varying lengths of time, and/or retraction of the published work.

Name: Xiangling Wang
Manuscript ID: JASN-2025-000496R1
Manuscript Title: Clinical Utility of Genetic Testing in Adults with Chronic Kidney Disease
Date of Completion: August 22, 2025
Disclosure Updated Date: August 22, 2025

ASN American Society of Nephrology | JASN Journal of the American Society of Nephrology | KIDNEY360 Accessing Our World From Every Angle | CJASN Clinical Journal of the American Society of Nephrology

ASN Journal Disclosure Form

As per ASN journal policy, I have disclosed any financial relationships or commitments I have held in the past 36 months as included below. I have listed my Current Employer below to indicate there is a relationship requiring disclosure. If no relationship exists, my Current Employer is not listed.

M. Westemeyer reports the following:
Employer: Natera, Inc.; and Ownership Interest: Natera, Inc.

I understand that the information above will be published within the journal article, if accepted, and that failure to comply and/or to accurately and completely report the potential financial conflicts of interest could lead to the following: 1) Prior to publication, article rejection, or 2) Post-publication, sanctions ranging from, but not limited to, issuing a correction, reporting the inaccurate information to the authors' institution, banning authors from submitting work to ASN journals for varying lengths of time, and/or retraction of the published work.

Name: Margaret Westemeyer
Manuscript ID: JASN-2025-000496R1
Manuscript Title: Clinical Utility of Genetic Testing in Adults with Chronic Kidney Disease
Date of Completion: August 14, 2025
Disclosure Updated Date: August 14, 2025

ASN
American Society of Nephrology
JASN
Journal of the American Society of Nephrology
KIDNEY360
Accessing Our World From Every Angle
CJASN
Clinical Journal of the American Society of Nephrology

ASN Journal Disclosure Form

As per ASN journal policy, I have disclosed any financial relationships or commitments I have held in the past 36 months as included below. I have listed my Current Employer below to indicate there is a relationship requiring disclosure. If no relationship exists, my Current Employer is not listed.

Z. Zhang reports the following:
Employer: Natera; and Ownership Interest: Natera.

I understand that the information above will be published within the journal article, if accepted, and that failure to comply and/or to accurately and completely report the potential financial conflicts of interest could lead to the following: 1) Prior to publication, article rejection, or 2) Post-publication, sanctions ranging from, but not limited to, issuing a correction, reporting the inaccurate information to the authors' institution, banning authors from submitting work to ASN journals for varying lengths of time, and/or retraction of the published work.

Name: Zhiji Zhang
Manuscript ID: JASN-2025-000496R1
Manuscript Title: Clinical Utility of Genetic Testing in Adults with Chronic Kidney Disease
Date of Completion: August 1, 2025
Disclosure Updated Date: August 1, 2025
