# Supplementary material for: Genetic Testing in the Management of Adult CKD
Source: J Am Soc Nephrol. 2025 Oct 29;37(4):777–89. doi: 10.1681/ASN.0000000913 (PMC13065169; doi:10.1681/ASN.0000000913)

SUPPLEMENT

Clinical Utility of Genetic Testing in Adults with CKD by Fouad T. Chebib et al.

1) Changes in Genetic Test Results After the Initial Test Report
2) Exemplary management changes by clinical category
3) Additional examples of clinical utility
4) Examples of Clinical Utility for Genetic Testing Among Patients with a Complimentary Kidney Biopsy
5) Supplemental Table 1: Study inclusion and exclusion criteria.
6) Supplemental Table 2: Physician One-year Post-test Questionnaire
7) Supplemental Table 3: Demographics for the full 1,388 patient cohort.
8) Supplemental Table 4: Management and prognosis changes based on genetic test results
9) Supplemental Table 5: Genetic findings in patients with multiple genetic findings
10) Supplemental Table 6: Pre- and post-test diagnoses for 29 patients with a complementary kidney biopsy.
11) Supplemental Table 7: FDA-approved treatments for patients with conditions covered by the Renasight panel.
12) Supplemental Table8: US Phase I-III trials for target CKD conditions
13) Supplemental Figure 1: Proportion of patients with positive or negative test findings whose genetic testing influenced clinical management and/or led to changes in management, stratified by clinical diagnosis.
14) Supplemental Figure 2: Proportion of patients with positive or negative test findings whose physician reported that genetic testing led to a change in their expected five-year prognosis, stratified by clinical diagnosis.

CHANGES IN GENETIC TEST RESULTS AFTER THE INITIAL TEST REPORT

At the study close, genetic testing results were compared with those at the original report date to determine the number and nature of cases with updated genetic test results. (e.g., establishment of new gene-disease associations, or upgrade of variant/s classified as a variant of uncertain significance (VUS) to a likely pathogenic/pathogenic variant). Following testing, 21 (1.5%) additional cases within the cohort of 1388 patients were changed to a new positive gene result. No positive results were downgraded. These genes included the following six genes: *ALG8* (n=3), *COL4A3* (n=1), *IFT140* (n=9), *PKD1* (n=6), *PKD2* (n=1), and *RRM2B* (n=1). For the variants in *IFT140* and *ALG8,* new evidence associated single *ALG8* and *IFT140* loss of function variants with ADPKD. These findings led to a result change from carrier status

(previously associated with an autosomal recessive ciliopathy) to a positive gene finding for 12 individuals. For the other nine individuals, incorporation of new data resulted in upgrades from VUS to pathogenic or likely pathogenic variant classifications. These findings comprised 6.3% of positive genetic test results at the close of the study. One-year questionnaire data for these patients was not included in the analysis as the timing between questionnaire completion and result upgrades was variable.

EXEMPLARY MANAGEMENT CHANGS BY CLINICAL CATERGORY

Cystic Nephropathy

Of 255 patients with a pre-test diagnosis of cystic nephropathy, 144 (56.5%) had positive genetic findings involving 16 genes (**Table 2**). Most (91.0%; 131/144) had variants in *PKD1* or *PKD2*, consistent with typical ADPKD; the remainder carried pathogenic variants in minor ADPKD-associated genes (*ALG8, ALG9, IFT140)* or other genes such as *OFD1 or PRKCSH ,* which may result in a PKD-like phenotype in some patients. Among 212 with follow-up data, 92.4% of those with a positive result had a reported change in clinical management.

Prognostic reassessments occurred in 72.9% (86/118) of positive cases: 37.2% were reclassified with a more stable expected disease course, often associated with minor ADPKD genes, while 62.8% were projected to progress more rapidly, frequently linked to truncating *PKD1* variants. Extra-renal features or specialist referrals (e.g. for cardiac anomalies or aneurysm screening) were noted in 14.4%. Thirteen individuals with *PKD1* or *PKD2* variants were on tolvaptan, with additional cases reporting discussions or changes in tolvaptan initiation or discontinuation. Lifestyle modifications were recommended in 11.9% of positive cases versus 1.1% of negative cases. Genetic testing also prompted family testing or reproductive counseling in 67.8% of positive cases compared to 14.9% of negative cases.

In ADPKD, all 55 physicians who initially reported a treatment change at one month affirmed the continued impact of the genetic findings at one year. Notable actions included diagnostic revision (n=8), initiation or consideration of tolvaptan (n=9), discontinuation of tolvaptan based on genetic result (n=2), and implementation of conservative management (n=1).

Proteinuric disease suggestive of a primary glomerulopathy

Among 236 patients with suspected primary glomerulopathy, 42 (17.8%) had positive findings across 13 genes (**Table 2**). At one year, 89.2% of these cases were reported to have had management changes influenced by genetic results. Prognosis was revised in 51.4%, with 63.2% indicating higher risk for relapse or faster progression. Specialist referrals for extra-renal manifestations (e.g., hearing loss, ocular disease) occurred in 13.5% of positive cases versus none in the negative group. Family counseling or cascade testing was initiated in 51.4% of positive cases (vs. 2.7% in negative), and lifestyle changes were recommended in 2.7% vs. 0.5%, respectively.

In a subgroup of patients with a one-year clinical diagnosis of *APOL1*-mediated kidney disease (n=16), 15 physicians elected conservative management, with one explicitly citing the genetic result as the reason. None of these patients received immunosuppressive therapy by one year, and one clinician reported discontinuing such treatment. Among 11 individuals with biopsy-confirmed FSGS, conservative management was chosen in 7 cases, including 2 where the decision was directly influenced by the genetic results. No new immunosuppressive therapy was initiated in this group, and 3 patients had treatment discontinued, with one physician attributing this decision to the genetic findings.

Of 61 patients with *COL4A3*, *COL4A4*, or *COL4A5* variants, 51 (83.6%) physicians reported that genetic findings were helpful for management at one year, and conservative therapy was initiated solely due to the genetic result in four. Transplant-related decisions, including donor

evaluation and selection, were influenced in 14 cases. RAAS inhibition or SGLT2 blockade was appropriately used in 12 of 32 patients with proteinuria. Extra-renal features attributed to genetic findings were identified in eight individuals.

Hypertension-Associated Nephropathy

Of 139 patients with presumed hypertensive nephropathy, 26 (18.7%) had positive genetic findings (**Table 2**). Among the 20 with follow-up data, 70% reported a change in management. Prognostic revision occurred in 50% of positive cases versus 17.7% of negative cases. A more rapid progression to ESKD was anticipated in 40% of positive versus 5.9% of negative cases. Referrals for extra-renal evaluation (e.g. retinal screening for *COL4A1*; liver evaluation for *NPHP1*) were made in 20% of positive cases. Family testing was initiated in 50% of positive vs. 4.2% of negative cases. Lifestyle recommendations were reported in 15% of positive cases, none among negative cases.

End-Stage Kidney Disease (ESKD)

Among 178 patients with ESKD, 26 (14.6%) had positive genetic test results spanning 13 genes, including *APOL1, COL4A3/4, TTR, PKD1, PKD2, CFH,* and others. Genetic testing was considered helpful or led to management change in 87.1% of these cases. Prognostic shifts were noted in 26.1% of positive and 21.9% of negative cases, though most indicated stable kidney function in both groups. Extra-renal evaluation or specialist referral was triggered in 8.7% of positive cases vs. 0% of negatives, likely influenced by the prevalence of syndromic genes in this group.

CKD of Unknown Etiology

Among 131 patients with unexplained CKD, 25 (19.1%) had positive results across 16 genes (Table 2). Of these, 68.4% were reported to have benefited from genetic testing through improved management. Prognosis was revised in 31.6% of positive cases and 19.0% of negatives. Among those with changed prognosis, a stable or slow progression was anticipated in 66.7% of positive cases and 93.8% of negatives.

Diabetes-Associated Nephropathy

Of 160 patients with presumed diabetic kidney disease, 16 (10.0%) had pathogenic variants across 11 genes (Table 2). Genetic findings were helpful or led to management change in 63.6% of positive cases. Prognosis changed in 36.4% of positive cases, 75% of which anticipated a more favorable outcome post-testing, versus 18.8% of negative cases. Family testing was reported in 9.1% of positive and 3.4% of negative cases. Lifestyle advice was provided in 9.1% of positive cases versus 2.6% in negative cases.

ADDITIONAL EXAMPLES OF CLINICAL UTILITY

Classification and Reclassification of Clinical Diagnosis

Positive genetic testing results led to a change in pre-test clinical diagnosis within one year in 28.9% (97/335) of patients. In the CKD of unknown etiology cohort (n=131), 25 (19.1%) individuals had positive genetic findings across 16 genes, highlighting the utility of genetic testing in resolving diagnostic uncertainty (**Table 2**). Among 160 patients with presumed diabetic nephropathy, 16 (10%) had positive results implicating genes such as APOL1, COL4A3, IFT140,

and SCNN1B (**Table 2**). These findings suggest that diabetes may not have been the primary etiology of CKD in a subset of cases. Similarly, in patients with a pre-test diagnosis of hypertensive nephropathy (n=139), 26 had positive genetic findings (**Table 2**); the results suggested a monogenic cause of CKD, indicating that hypertension was likely secondary in 18.7% of cases (26/139) and not the primary cause of CKD. Among 61 individuals with at least one COL4A3/4/5 variant, 38 (62.2%) were reported to have had a change in diagnosis based on the genetic result. In 14 individuals with 1-year clinical diagnosis of APOL1-mediated kidney disease, clinicians reported pre-test diagnosis change in 6 (42.9%) of patients because of genetic test results. Finally, among four individuals initially diagnosed with an electrolyte and/or acid–base disorder, genetic testing refined the diagnosis to Bartter or Gitelman syndrome in two cases.

Negative genetic test results:

Additional examples of management changes in individuals with negative genetic findings, beyond those described in the main text, include the following: among 85 individuals with a one-year clinical diagnosis of proteinuric disease suggestive of a primary comprising FSGS (n = 60; primary: 5, secondary: 19, unspecified: 41), proteinuria suggestive of primary glomerulopathy (n = 15), and other glomerular disorders, change in therapy (initiation of immunosuppressive therapy or conservative therapy or discontinuation of other therapy) was documented in 7 cases (8.2%) **because** of the negative genetic test results. These are conditions in which steroid treatment would generally not be indicated if a genetic diagnosis were confirmed.

EXAMPLES OF CLINICAL UTILITY FOR GENETIC TESTING AMONG PATIENTS WITH A COMPLEMENTARY KIDNEY BIOPSY

Among the 21 cases that had histological features of focal segmental glomerulosclerosis (FSGS) on biopsy, 90.5% (n=19) also had corroborative gene results. In one case, a patient who presented with hematuria and a normal eGFR, histology from the kidney biopsy showed nonspecific findings of “thin glomerular basement membrane that was segmental, with mild wrinkling.” Ultimately, genetic testing showed a positive finding in *CUBN*, clarifying the diagnosis as chronic proteinuria. In another case, a 56-year-old with ESKD who had a diagnosis of diabetes mellitus type 2 for 20 years underwent a kidney biopsy with histology that was consistent with nephropathy associated with diabetes. Genetic testing revealed a positive finding in *HNF1A* reclassifying this disease to maturity-onset diabetes of the young (MODY) which has different management implications.

Several potential emerging associations between histologic findings and molecular genetic diagnoses were noted. Five patients in this cohort with IgA nephropathy identified through biopsy were found to have positive gene findings that may clarify the diagnosis and impact the management. The gene findings included: a patient with both *APOL1* and *HBB*, two patients with a single variant in *COL4A4*, one patient with a *COL4A5* variant, and a patient with *SLC12A3* positive finding. Another emerging and novel finding from these data showed the presence of membranous nephropathy on histology in two patients with *APOL1* positive gene findings.

Uninformative/Nonspecific Histology:

- Five additional cases had biopsy findings described as nonspecific or inconclusive.

We believe that genetic testing performed prior to kidney biopsy could, in selected scenarios, reduce the need for an invasive procedure. Several cases in our cohort support this possibility:

• In the aforementioned CUBN and HNF1A cases, genetic testing yielded critical diagnostic insights that were not apparent from histology.

• Among patients with FSGS, a strong correlation was observed between genetic findings and histology (90.5% concordance), suggesting that pre-biopsy genetic testing in individuals with suspected hereditary FSGS could guide clinical management and, in some cases, may obviate biopsy.

• Similarly, the detection of COL4A4/COL4A5 variants in patients with minimal biopsy findings or ambiguous diagnoses (e.g., IgA nephropathy) suggests that molecular diagnoses may help refine or even supersede histologic interpretations.

However, we also emphasize that biopsy remains a valuable tool in the nephrologist's diagnostic arsenal, particularly when:

• Multiple pathologies are suspected,

• Genetic testing is uninformative or inconclusive, or

• Histologic assessment is required for disease staging or therapeutic decision-making.

We propose that a sequential or integrated diagnostic approach, incorporating both histologic and genetic information, offers the most comprehensive framework for patient care, balancing diagnostic yield with procedural risk.

Supplemental Table 1: Study inclusion and exclusion criteria

Inclusion criteria

—-------------------

- 18 years and older at time of signing the informed consent
- For patients 65 years and older: absence of family history of CKD or suspicion of genetic etiology of kidney disease
- Diagnosis kidney disease, and/or one of the following:
  - Kidney disease not otherwise specified
  - Nephropathy associated with diabetes mellitus
  - Nephropathy associated with hypertension
  - Cystic nephropathy
  - Chronic kidney disease of unknown cause after standard nephrological evaluation
  - End stage renal disease
  - Congenital nephropathy
  - Tubulointerstitial disease of unknown etiology
  - Proteinuria disease suggestive of a primary glomerulopathy
  - Hematuria
  - Early onset, severe, or familial hypertension
  - Thrombotic microangiopathy
  - Electrolyte and/or acid-base disorder
  - Nephrolithiasis with family history
- Able to read, understand, provide written informed consent
- Willing and able to comply with the study-related procedures

Exclusion criteria

—-----------------------

- History of kidney transplant
- Clinical features and kidney biopsy diagnosis strongly indicative of a secondary nephropathy (e.g., diabetic nephropathy, lupus nephritis, acute kidney injury)
- Previously confirmed diagnosis of a monogenic etiology of kidney disease from previous genetic testing
- Receipt of blood transfusion within 30 days of study blood draw

Supplemental Table 2: Physician One-year Post-test Questionnaire

Natera_20_048_TRP_Post-Test Physician Questionnaire (Y1_Y2_Y3)

☐1 Year ☐2 Year ☐3Year

1) Was genetic testing helpful for clinical management of this patient, regardless of the Renasight genetic test results (negative or positive)?

- o Y
- o N

2) What is your patient's clinical diagnosis? ______________________________

3) Did the patient's pre-test diagnosis change because of the Renasight genetic test results?

- o Y
- o N

4) Have you done one of the following over the past year?

	Not done	Done because of genetic test results	Done independent of genetic test results
a. Initiated conservative therapy only (e.g., renin angiotensin blockade, blood pressure control, diet, etc.)			
b. Initiated SGLT2 inhibition			
c. Initiated immunosuppression with steroids			
d. Initiated immunosuppression with other agents (e.g., calcineurin inhibitors, MMF, etc.)			

e. Initiated a different treatment not mentioned above			
If done, specify what additional treatment (not mentioned above) was initiated:	Text		
f. Discontinued immunosuppression			
g. Discontinued other medication(s)			
If done, specify what other medication(s) were discontinued:	Text		
h. Referred for dialysis			
i. Referred for transplantation			
If done, indicate recommendation:	Genetic testing for the kidney donor<br><br>Screening the patient's relatives for the genetic Disorder<br><br>Choosing a different kidney donor		
j. Recommend lifestyle modification			
k. Identified extra-renal clinical features			
l. Referred to non-kidney specialists (i.e., Ophthalmologist, Otolaryngologist, etc.)			

If done, indicate which specialists the patient was referred to:	Cardiology<br>Ophthalmology<br>Head and Neck Surgery/Otology<br>Neurology/Neurosurgery<br>Hepatology/GI<br>Urology<br>Other		
If Other, Specify	Text		
m. Referred to specialized renal center (e.g., Genetics, Alport Center of Excellence)			
n. Ordered additional imaging/diagnostic workup			
If done, indicate which additional imaging or diagnostic workup was ordered:	Radiology<br>Biopsy<br>Lab Work<br>Other		
If Lab Work, Specify:	Text		
If Other, Specify:	Text		
o. Referred to clinical trial enrollment			
p. Recommended family testing/risk assessment			
q. Other recommendations			
If done, indicate what other treatment, management, or recommendations were made:	Text		

5. **Has the prognosis for this patient in the next five (5) years changed because of the Renasight test result? (*Check all that apply*)**

❑ No

- ❑ Yes, remission of current active disease is expected
- ❑ Yes, possible relapsing of kidney disease is a concern
- ❑ Yes, stable kidney function is expected
- ❑ Yes, slower progression to ESRD is expected
- ❑ Yes, rapid progression to ESRD is expected

6. Did the experience with this patient change your interest in genetic testing? (*Check all that apply*)

- ❑ Yes, I will discuss genetic testing with more patients
- ❑ Yes, I will refer my patients to genetic counseling more often
- ❑ Yes, I will order genetic testing **more** often
- ❑ Yes, I will order genetic testing **less** often
- ❑ No, it did not change my interest **in referring my patients for genetic counseling**
- ❑ No, it did not change my interest **in ordering genetic testing for my patients**

Supplemental Table 3: Full Cohort demographics, including all patients active in the study at the 1-year timepoint.

	All Patients (N = 1388)	Positive (N = 335)	Negative (N = 1053)	p-value[1]	FDR adjusted p-value[2]
**Age (yrs)**					
Mean (SD)	51.2 (14.4)	46.7 (14.4)	52.6 (14.1)	<0.001[3]	<0.001
**Age Groups**					
18-39 yo	306 (22.0%)	106 (31.6%)	200 (19.0%)	<0.001[4]	<0.001
40-64 yo	849 (61.2%)	185 (55.2%)	664 (63.1%)	0.012[4]	0.021
65+ yo	233 (16.8%)	44 (13.1%)	189 (17.9%)	0.044[4]	0.066
**Sex at Birth**				0.005[4]	0.011
Female	692 (49.9%)	190 (56.7%)	502 (47.7%)		
Male	696 (50.1%)	145 (43.3%)	551 (52.3%)		
**Race**					
White	912 (65.7%)	221 (66.0%)	691 (65.6%)	>0.9[4]	>0.9
Black/African-American	236 (17.0%)	73 (21.8%)	163 (15.5%)	0.009[4]	0.019
Asian	76 (5.5%)	13 (3.9%)	63 (6.0%)	0.2[4]	0.2
Native/Alaskan/Hawaiian/Pacific	21 (1.5%)	2 (0.6%)	19 (1.8%)	0.13[4]	0.2
Other/Unknown/Not Reported	156 (11.2%)	29 (8.7%)	127 (12.1%)	0.092[4]	0.12
**Ethnicity**				0.002[4]	0.005
Hispanic or Latino	334 (24.1%)	59 (17.6%)	275 (26.1%)		
Not Hispanic or Latino	1,033 (74.4%)	271 (80.9%)	762 (72.4%)		
Unknown	21 (1.5%)	5 (1.5%)	16 (1.5%)		

**Family History of CKD**				<0.001[4]	<0.001
No	949 (68.4%)	183 (54.6%)	766 (72.7%)		
Yes	434 (31.3%)	152 (45.4%)	282 (26.8%)		
Not Reported	5 (0.4%)	0 (0.0%)	5 (0.5%)		

[1]Statistical test p-value comparing positive result patients with negative/VUS result patients

[2]False discovery rate correction for multiple testing

[3]Wilcoxon rank sum test

[4]Fisher's exact test

Supplemental Table 4: Management and prognosis changes based on genetic test results

	Overall	Positive (P/LP) Results	Negative Results
Genetic testing was helpful or led to a change in clinical management	52.5% 616/1174	86.5% 237/274	42.1% 379/900
Family test or risk assessment recommendation	15.4% 181/1174	53.6% 147/274	3.8% 34/900
Identified extra-renal features or referred to specialist	3.8% 45/1174	14.2% 39/274	0.7% 6/900
Lifestyle changes recommendation	2.9% 34/1174	8.4% 23/274	1.2% 11/900
Genetic testing led to a change in prognosis	26.7% 314/1174	55.1% 151/274	18.1% 163/900
Prognosis worsened	34.4% 108/314	51.0% 77/151	19.0% 31/163
Prognosis improved	66.6% 209/314	50.3% 76/151	81.6% 133/163

Supplemental Table 5: Genetic findings in patients with multiple genetic findings

*APOL1, ABCC8**	*APOL1, COL4A4**	*CFH*, TRPC6**	*COL4A4*, KANSL1*
APOL1, HBB (2)*	*CASR*, SALL1*	*COL4A4, PKD1**	*PKD2, SLC4A1*
APOL1, MC4R*	*COL4A3, PKD1**	*PKD1*, SLC7A9*	*APOL1, COL4A3*, COL4A4**
*APOL1, SMAD9**	*COL4A3, SLC12A3**	*PKD1*, TRPC6*	*APOL1, CYP17A1, PKD1**
APOL1, TTR*	*COL4A4*, COL4A5**	*PKD1*, TTR*	*APOL1*, SLC3A1, TTR*

*gene variant most likely to account for the majority of the clinical features.

Supplemental Table 6: Pre- and post-test diagnoses for 29 patients with a complementary kidney biopsy

Pre-Test Primary Clinical Diagnosis	Positive Gene Finding	Gene:disease association	Number of patients
Hematuria	*CUBN*	Proteinuria, chronic benign; Megaloblastic Anemia 1, FinnishType	1
Nephropathy associated with Hypertension	*HNF1A*	HNF1A-MODY (Maturity-Onset Diabetes of the Young)	1
Proteinuric disease suggestive of a primary glomerulopathy	*APOL1*	Susceptibility to End-Stage Renal Disease & Focal Segmental Glomerulosclerosis 4	11
Proteinuric disease suggestive of a primary glomerulopathy	*APOL1* *COL4A3* *COL4A4*	Susceptibility to End-Stage Renal Disease & Focal Segmental Glomerulosclerosis 4/ COL4A3-related Alport syndrome/ COL4A4-related Alport syndrome	1
Proteinuric disease suggestive of a primary glomerulopathy	*APOL1* *COL4A4*	Susceptibility to End-Stage Renal Disease & Focal Segmental Glomerulosclerosis 4/ COL4A4-related Alport syndrome	1
Proteinuric disease suggestive of a primary glomerulopathy	*APOL1* *HBB*	Susceptibility to End-Stage Renal Disease & Focal Segmental Glomerulosclerosis 4/ Beta-Hemoglobinopathies; Sickle cell disease	1
Proteinuric disease suggestive of a primary glomerulopathy	*COL4A3*	COL4A3-related Alport syndrome	2
Proteinuric disease suggestive of a primary glomerulopathy	*COL4A4*	COL4A4-related Alport syndrome	3
Proteinuric disease suggestive of a primary glomerulopathy	*COL4A5*	Alport syndrome, X-linked	2
Proteinuric disease suggestive of a primary glomerulopathy	*HBB*	Beta-Hemoglobinopathies (AD/AR); Sickle cell disease (AR)	1
Proteinuric disease suggestive of a primary glomerulopathy	*NPHS2*	Congenital nephrotic syndrome, type 2	1
Proteinuric disease suggestive of a primary glomerulopathy	*SMARCAL1*	Schimke immunoosseous dysplasia	1
Proteinuric disease suggestive of a primary glomerulopathy	*WAS*	Wiskott-Aldrich Syndrome, X-Linked Thrombocytopenia (XL); X-Linked Severe Congenital Neutropenia (XL)	1
Tubulointerstitial disease of unknown etiology	*FAN1*	Interstitial Nephritis, Karyomegalic	1
Tubulointerstitial disease of	*NPHP1*	Nephronophthisis 1, Juvenile; Senior-Loken	1

unknown etiology		Syndrome; Joubert Syndrome 4	
Total			29

Supplemental Table 7: FDA-approved treatments for patients with conditions covered by the Renasight panel.

Gene	Condition	Generic Name	Therapy—Manufacturer	Mechanism of Action
PKD1	Polycystic Kidney Disease	Tolvaptan	Jynarque™—Otsuka	Vasopressin V2 antagonist
GLA	Fabry Disease	Agalsidase alfa<br>Migalastat hydrochloride	Replagal™—Takeda<br>Galafold™—Amicus	α-galactosidase A replacement or<br>α-galactosidase A chaperone
JAG1, NOTCH2	Alagille Syndrome	Odevixibat<br>Maralixibat chloride	Bylvay™—Albiero<br>Livmarli™—Mirum	Ileal bile acid transporter antagonist
TTR	Hereditary Amyloidosis	Acoramidis hydrochloride<br>Eplontersen<br>Vutrisiran<br>Patisiran<br>Inotersen<br>Tafamidis	Attruby™—BridgeBio Pharma<br>Wainua ™— Ionis Pharma<br>Amvuttra™—Alnylam<br>Onpattro™—Alnylam<br>Tegsedi™—Ionis Pharma<br>Vyndamax™—Pfizer	Transthyretin stabilization or<br>Transthyretin production inhibition
ARL6, BBS1, BBS10, BBS12, BBS2, BBS4, BBS5, BBS7, BBS9, C8orf37, LZTFL1, MKKS, SDCCAG8, TTC8, WDPCP	Bardet-Biedl syndrome	Setmelanotide	Imcivree™— Rhythm Pharmaceuticals	Melanocortin-4 receptor agonist
CTNS	Cystinosis	Cysteamine bitartrate	Procysbi™—Amgen	Enhancing cysteine excretion
SLC3A1, SLC7A9	Cystinuria	Tiopronin	Thiola™— Travere	Enhancing cysteine excretion

MEFV	Familial Mediterranean Fever	Canakinumab Colchicine	ILaris™— Novartis Colcrys™— Takeda	Interleukin-1 beta antibody or Neutrophil chemotaxis inhibition
C3, CFH, CFHR5, CFI, DGKE, THBD	Atypical Hemolytic Uremic Syndrome (aHUS)	Eculizumab Ravulizumab	Solaris™—AstraZeneca Ultomiris™— AstraZeneca	Complement protein C5 inhibition
AGXT	Primary hyperoxaluria, Type 1	Lumasiran Nedosiran	Oxlumo™—Alnylam Rivfloza™ —Novo Nordisk	RNA interference targeting glycolate oxidase or hepatic lactate dehydrogenase
ALPL	Hypophosphatasi a	Asfotase alfa	Strensiq™— AstraZeneca	Alkaline phosphatase enzyme replacement
DLC1, LAMB2, MAGI2, PLCE1, PTPRO, TNS2	Nephrotic Syndrome	Cyclosporine Corticotropin Rituximab	Neoral™—Novartis Acthar™—Mallinckrodt Rituxin™ — Biogen	Immunosuppressio n; Enhancing glomerular permeability; Enhancing glucocorticoid production; B-cell depletion

Supplemental Table 8: US Phase I-III trials for target CKD conditions [data as of 2/20/25]

Target Conditions	Trial Phase	# of Trials
• Alport Syndrome<br>• Atypical Hemolytic Uremic Syndrome<br>• C3 Glomerulopathy<br>• Cystinuria<br>• Fabry Disease<br>• Alagille Syndrome<br>• Cystinosis<br>• Polycystic Kidney Disease<br>• Renal Disease	Phase I	11
	Phase I/II	10
	Phase II	45
	Phase II/III	7
	Phase III	39

Supplemental Figure 1: Proportion of patients with positive or negative test findings whose genetic testing influenced clinical management and/or led to changes in management, stratified by clinical diagnosis.

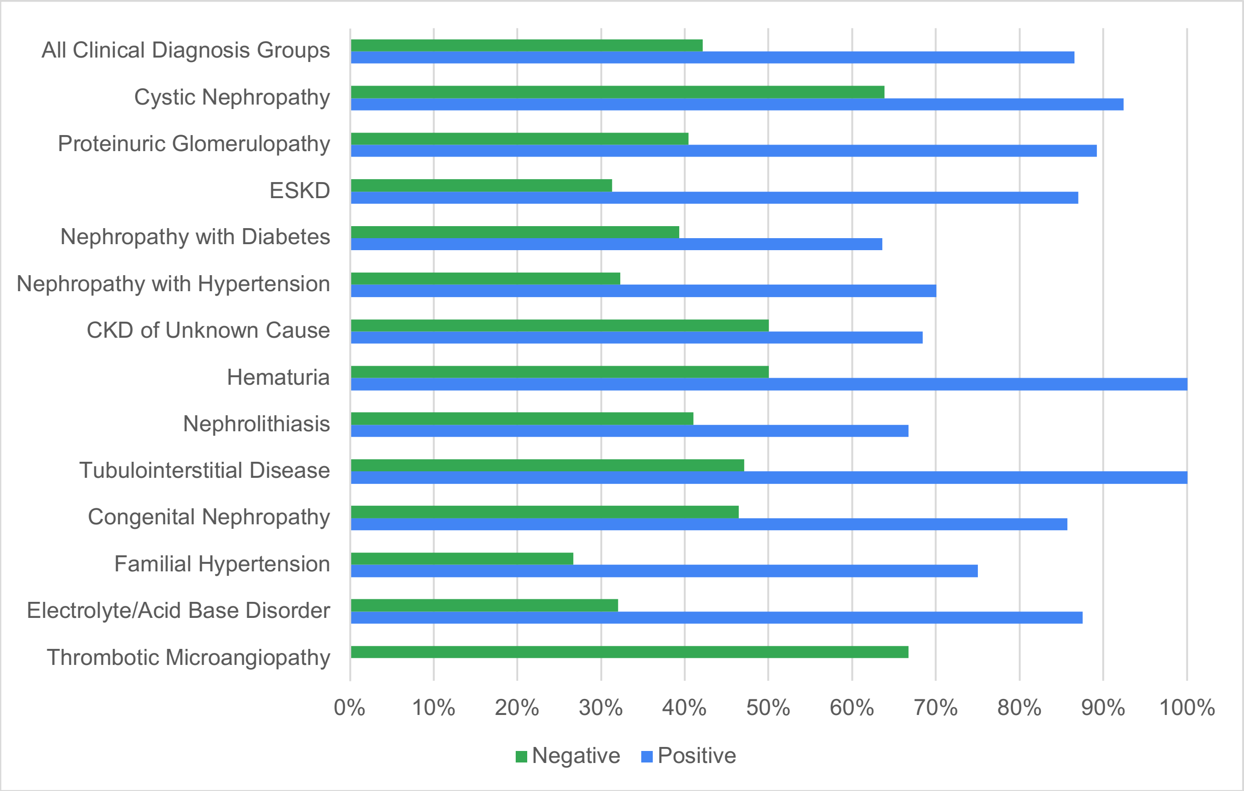

Supplemental Figure 2: Proportion of patients with positive or negative test findings whose physician reported that genetic testing led to a change in their expected five-year prognosis, stratified by clinical diagnosis.

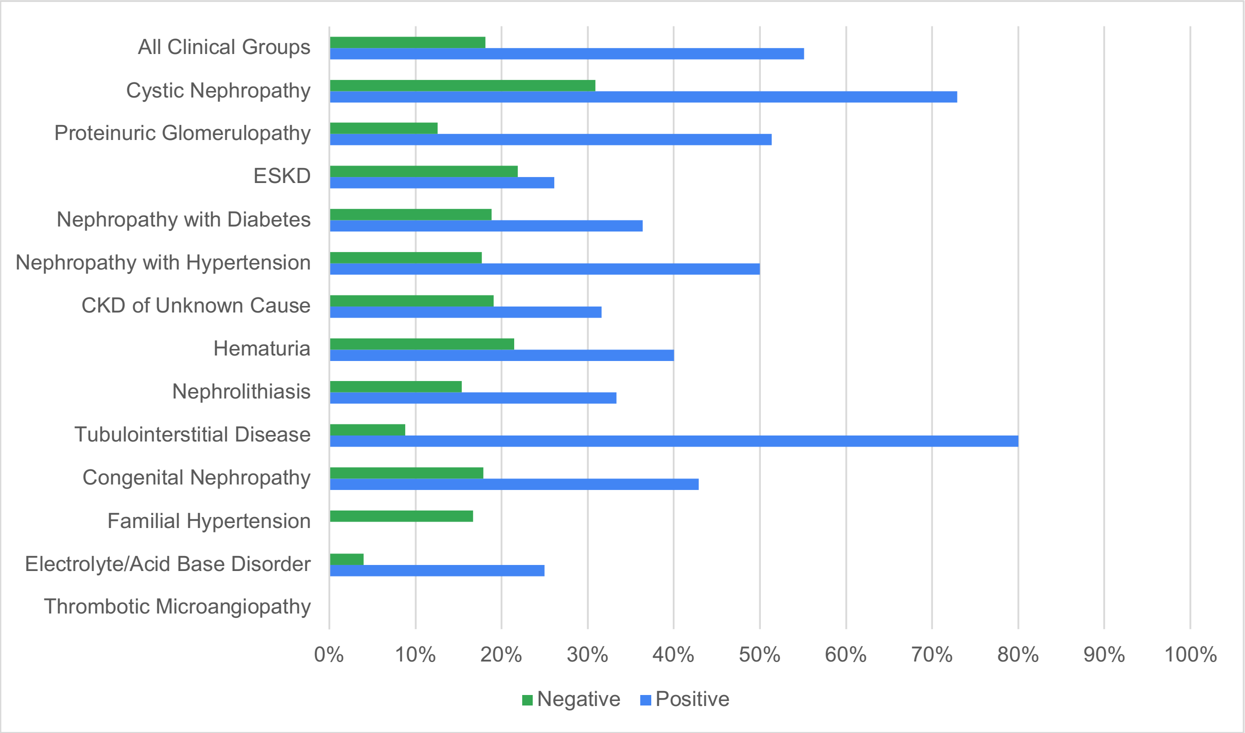

Supplement: Supplementary file 2 [file jasn-37-777-s002.pdf]
